# Large Oncosomes Induce Pro‐Metastatic Stromal‐Myeloid Reprogramming

**DOI:** 10.1002/advs.78406

**Published:** 2026-10-25

**Authors:** Taylon Felipe Silva, Virginia Marcia Concato‐Lopes, Fatima Bedier, Lauren Newman, Diana Kitka, Jamelle Brown, Blandine Victor, Minhyung Kim, Tatyana Vagner, Catherine Grasso, Dmitriy Sheyn, Sungyong You, Michael R. Freeman, Fayyaz S. Sutterwala, Helen S. Goodridge, Caroline Jefferies, Paola de Candia, Jlenia Guarnerio, Dolores Di Vizio

**Affiliations:** ^1^ Department of Urology Cedars‐Sinai Health Sciences University Los Angeles California USA; ^2^ Department of Medicine Cedars‐Sinai Health Sciences University Los Angeles California USA; ^3^ Department of Biomedical Sciences Cedars‐Sinai Health Sciences University Los Angeles California USA; ^4^ Department of Orthopaedics Cedars‐Sinai Health Sciences University Los Angeles California USA; ^5^ Department of Molecular Medicine and Medical Biotechnology University of Naples Federico II Naples Italy; ^6^ Department of Radiation Oncology Cedars‐Sinai Health Sciences University Los Angeles California USA; ^7^ Department of Genetics University of Texas MD Anderson Cancer Center Houston Texas USA; ^8^ Department of Radiological, Oncological and Pathological Sciences Sapienza University of Rome Rome Italy

**Keywords:** bone marrow microenvironment, innate immune sensing, large oncosomes, mesenchymal stem cells, metastasis, neutrophils, stromal reprogramming

## Abstract

Bone metastasis can be facilitated by systemic remodeling, yet the tumor‐derived signals that influence the bone marrow microenvironment remain incompletely understood. Large oncosomes (LOs) are atypically large (> 1 µm), tumor‐derived extracellular vesicles shed by amoeboid cancer cells. Here, we demonstrate that LOs function as systemic mediators of metastatic niche formation by activating innate immune sensing in bone marrow mesenchymal stem cells (BM‐MSCs). Systemic administration of LOs to immunocompetent tumor‐bearing mice did not affect primary tumor growth but increased metastatic burden. LOs induced a robust, dose‐dependent interferon‐driven inflammatory program, characterized by interferon‐stimulated genes and neutrophil chemokines. Mechanistically, this response was driven by LO‐associated nucleic acids activating convergent cytosolic DNA‐ and RNA‐sensing pathways in recipient stromal cells. LO‐conditioned BM‐MSCs promoted the accumulation and polarization of neutrophils toward an immunosuppressive, polymorphonuclear myeloid‐derived suppressor cell (PMN‐MDSC)‐like phenotype. In vivo, genetic suppression of LO shedding in tumor cells reduced PMN‐MDSC accumulation in the bone marrow, which was reversed by systemic LO administration. Together, these findings support a model in which LO‐associated signals reprogram the bone marrow microenvironment to support metastatic colonization, identifying a previously unrecognized mechanism linking tumor vesiculation to immune remodeling at distant sites.

## Graphical abstract

Large oncosomes systemically reprogram bone marrow mesenchymal stem cells through innate nucleic acid sensing, driving inflammatory and immunosuppressive remodeling that supports a metastasis‐permissive bone marrow environment, revealing a novel tumor‐host communication axis in cancer.

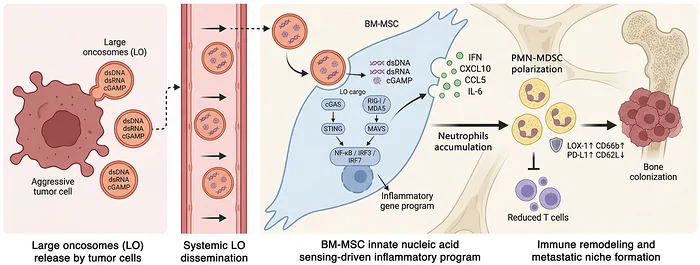

## 1 Introduction

Metastatic progression is increasingly recognized as a systemic process driven by continuous communication between tumor cells and distant host tissues [1, 2, 3]. Through this reciprocal crosstalk, primary tumors actively remodel metastatic sites even before the arrival of disseminated tumor cells, creating permissive microenvironments that support metastatic colonization [3, 4, 5]. Extracellular vesicles (EVs) have emerged as central mediators of this long‐range communication, coordinating diverse processes that include stromal activation, immune modulation, and organ‐specific niche formation [5, 6, 7, 8]. The expanding recognition of EV heterogeneity has further highlighted the need to define how distinct EV populations contribute to these processes [9, 10, 11].

Large oncosomes (LOs) represent a biologically distinct class of tumor‐derived EVs. LOs are atypically large (> 1 µm), membrane bleb‐derived vesicles selectively shed by highly migratory and invasive tumor cells with metastatic capacity [12, 13, 14]. Their biogenesis is tightly linked to cytoskeletal remodeling, non‐apoptotic, amoeboid membrane blebbing, and cellular programs associated with nuclear shape instability and highly malignant tumor phenotypes [12, 15, 16]. LOs are abundant in the circulation of patients with advanced malignancies [10, 12, 17, 18, 19, 20] and have been implicated in multiple aspects of tumor progression, including tumor cell adhesion, invasion, angiogenesis, and metastatic dissemination [12, 21, 22, 23, 24].

The biological function of LOs is closely linked to their diverse molecular cargo. Profiling studies, including comparative multiomics of LOs and small EVs (sEV), have revealed that LOs are enriched in diverse classes of tumor‐derived cargo, such as proteins, lipids, genomic DNA, and multiple RNA species [10, 13, 17, 22, 25, 26, 27, 28]. The abundance and diversity of nucleic acids within LOs support their investigation as carriers of signals that influence recipient‐cell behavior. A recent study has further implicated LOs in pre‐metastatic niche conditioning and organ‐specific metastatic tropism, extending their role beyond local tumor progression to systemic regulation of metastatic competence [23].

Tumor‐derived nucleic acids have emerged as regulators of stromal and immune cell behavior [29]. In both cancer and tissue injury, aberrant DNA and RNA species activate innate immune sensing pathways that converge on type I interferon as an inflammatory signaling program. This type of response can profoundly influence stromal cell states, myeloid cell recruitment, and the establishment of immunoregulatory microenvironments that support tumor progression and metastasis [30, 31, 32]. Cancer‐derived molecular signals, including mitochondrial DNA and other damage‐associated nucleic acids, have been shown to reprogram myeloid populations through innate immune mechanisms [31]. Similarly, LOs isolated from cancer stem cells have been reported to carry cytokines and other bioactive cargo capable of modulating myeloid cell behavior [33]. Together, these observations raise a fundamental question on the biology of metastasis: how are tumor‐derived inflammatory signals disseminated systemically to coordinate stromal and immune remodeling across distant tissues?

In this study, we investigated how tumor‐derived LOs contribute to systemic communication during metastatic progression using prostate cancer and breast cancer as two models of bone metastasis. Using integrated molecular, cellular, and in vivo approaches, we investigated how LO‐associated nucleic acids activate specific innate immune pathways in bone marrow stem cells, thereby promoting stromal and immune reprogramming linked to metastasis. Together, this work provides a novel mechanistic framework for understanding how LOs mediate tumor‐host communication during metastasis.

## 2 Results

### 2.1 LOs Enhance Tumor Cell Migration and Metastatic Colonization

Given the emerging role of tumor‐derived EVs in metastatic niche formation and systemic tumor‐host communication, we decided to investigate whether LOs and sEVs influence metastatic colonization in vivo. To address this question, we employed a syngeneic breast cancer model (4T1.2 cells) with bone metastatic propensity [34, 35]. Using a two‐phase “education” approach, immunocompetent BALB/c mice bearing orthotopic mammary fat pad tumors received systemic retro‐orbital administration of either LOs or sEVs derived from the 4T1.2 cell line throughout primary tumor progression (Figure 1A). The bone‐metastatic 4T1.2 model was used for the in vivo studies because its syngeneic implantation in BALB/c mice preserves the stromal–immune interactions relevant to LO‐associated remodeling.

**FIGURE 1:**
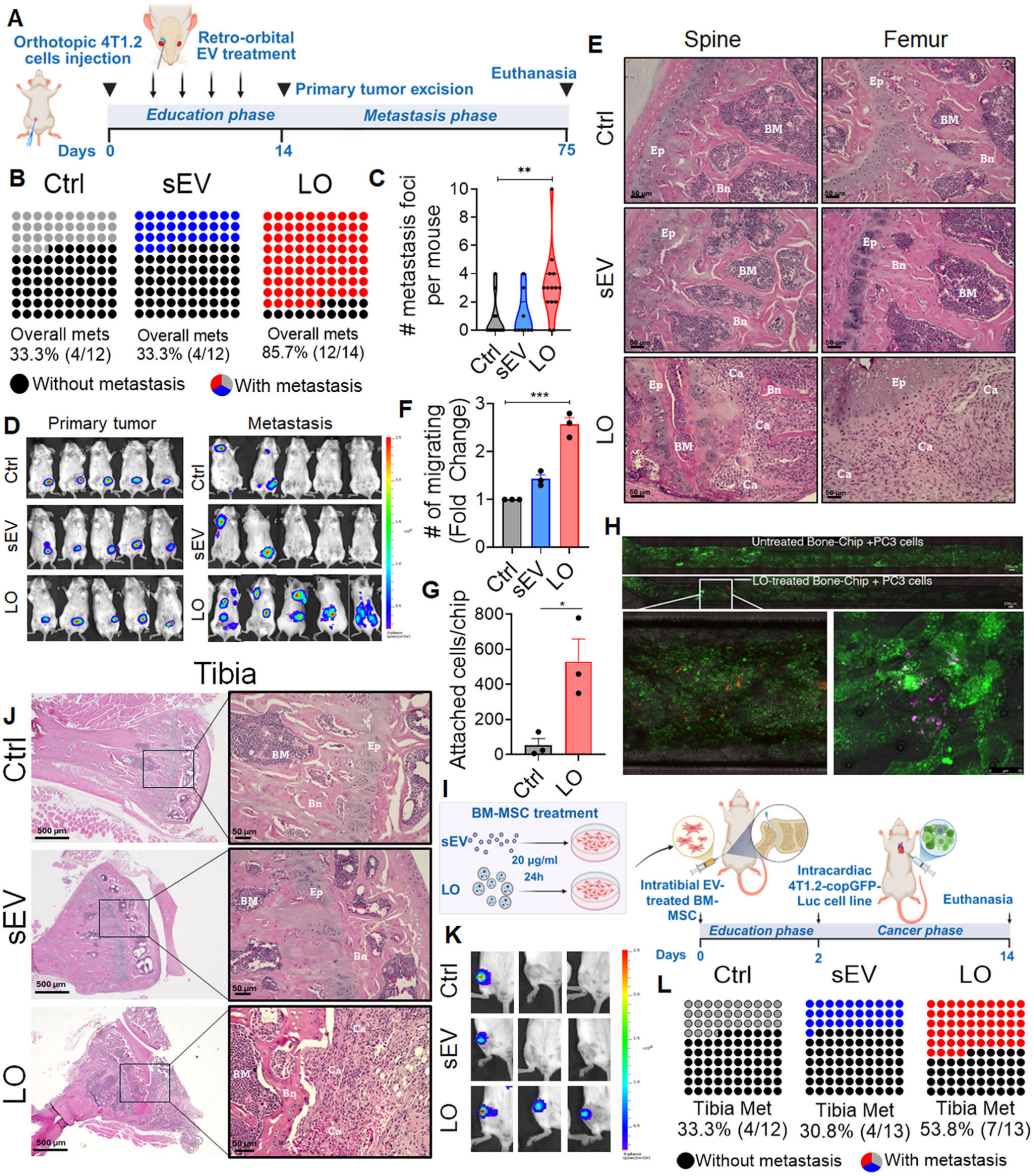
Large oncosomes (LOs) exhibit enhanced pro‐metastatic activity. (A) Schematic of the metastasis‐education model using orthotopic implantation of 1 × 10^5^ 4T1.2‐Luc/GFP cells in BALB/c mice followed by intravenous administration of 10 µg/injection of LO, small extracellular vesicles (sEV), or vehicle (education phase) derived from 4T1.2 cells. Primary tumors were excised and metastasis development evaluated for an additional 60 days (metastasis phase), *n* ≥ 14 mice per group. (B) Waffle plots showing metastatic incidence in the control‐ (Ctrl), sEV‐, and LO‐educated groups. (C) Quantification of metastatic foci in each experimental group. (D) Representative IVIS images of mice from each group at the end of the education phase (left) and at endpoint (60 days after primary tumor excision; right). (E) Hematoxylin and Eosin (H&E) staining of spine and femur sections. Scale bar 50 µm. (F) Transwell recruitment assay in which PC3 cells migrate toward conditioned medium from bone marrow‐derived mesenchymal stem cells (BM‐MSCs) treated with PC3‐derived LO, sEV, or Ctrl. (G, H) Quantification and imaging of the bone‐on‐a‐chip assay showing red fluorescence protein (RFP)‐PC3 (red) migration toward green fluorescence protein (GFP)‐MSCs (green) pre‐exposed to LO. Internalized RFP‐PC3‐derived LO is shown in the left panel (magenta), and quantification of the total number of cancer cells attaching to the BM‐MSC‐containing matrix is shown in the right panel. Scale bars: 200 and 25 µm. (I) Schematic of the BM‐MSC‐priming bone metastasis model: BALB/c BM‐MSCs pretreated for 24 h with 4T1.2‐derived LO, sEV, or vehicle were injected (2 × 10^4^) into the tibia of Balb/c. After 48 h, mice received intracardiac injection of 1 × 10^5^ 4T1.2‐Luciferase/GFP cells, and tibia colonization was analyzed after 14 days. n ≥ 12 mice per group. (J) H&E staining of tibia sections (scale bars 50 and 500 µm). (K) Representative in vivo imaging (IVIS) images of the knee region at endpoint. (L) Waffle plots showing metastatic incidence in tibias pre‐injected with MSCs treated with LO, sEV, and Ctrl. Ep – epiphysis; BM – bone marrow; Bn – bone; Ca – Cancer cells.

LO‐treated mice showed an increase in metastatic incidence, with metastases detected in 85.7% of animals compared with 33.3% in both control and sEV‐treated groups (Figure 1B). Consistent with this, LO education significantly increased the number of metastatic foci per mouse, indicating that LOs promote not only metastatic occurrence but also overall metastatic burden (Figure 1C). Importantly, this result was not explained by differences in primary tumor growth, as in vivo imaging (IVIS) showed comparable primary tumor signal across groups, while revealing broader metastatic dissemination in LO‐treated animals after primary tumor excision (Figure 1D). Histological analysis further supported these findings. Spine and femur sections from control and sEV‐treated mice showed largely preserved bone architecture, including defined epiphyseal regions, intact bone structures, and normal‐appearing bone marrow. In contrast, bones from LO‐treated mice displayed disrupted architecture, altered epiphyseal organization, reduced bone area consistent with the osteolytic behavior of this model, and extensive replacement of bone marrow by tumor cells (Figure 1E). Together, these data indicate that systemic exposure to LOs during primary tumor progression enhances metastatic colonization and bone involvement without increasing primary tumor growth.

The ability of LOs to enhance metastatic dissemination prompted us to examine their impact on stromal populations that regulate metastatic niche formation. We investigated whether LOs contribute to stromal education through the modulation of bone marrow‐derived mesenchymal stem cells (BM‐MSCs, which are known to play a key role in establishing a supportive niche for metastatic tumor cells [36, 37, 38]. LO‐treated BM‐MSCs significantly enhanced prostate cancer PC3 cells migration compared to ctrl and sEV in an in vitro transwell assay (Figure 1F). This was further confirmed using a dynamic 3D bone‐on‐a‐chip, where LO‐primed BM‐MSCs recruited substantially more prostate cancer cells than naïve BM‐MSCs (Figure 1G,H).

To determine whether LO‐conditioned stromal cells are sufficient to enhance metastatic colonization in vivo, BALB/c‐derived BM‐MSCs were pre‐treated with 4T1.2‐derived LOs or sEVs and injected intratibially into syngeneic BALB/c mice prior to intracardiac inoculation of 4T1.2 tumor cells (Figure 1I). This approach allowed us to test whether local stromal education by tumor‐derived vesicles creates a more permissive bone microenvironment for subsequent metastatic seeding. Histological analysis of the injected tibias showed preserved bone architecture in control and sEV‐primed groups, whereas tibias receiving LO‐conditioned BM‐MSCs displayed overt tumor infiltration and disruption of the bone marrow compartment (Figure 1J). Consistent with these findings, (IVIS imaging revealed focal metastatic signal in the injected tibial region of LO‐primed animals (Figure 1K). Quantification across the cohort showed that tibial metastases developed in 53.8% of mice receiving LO‐conditioned BM‐MSCs, compared with 33.3% and 30.8% of mice in the control and sEV‐conditioned BM‐MSC groups, respectively (Figure 1L). Together, these data suggest that LO‐mediated reprogramming of BM‐MSCs enhances the ability of the local bone microenvironment to support metastatic colonization.

### 2.2 LOs Induce an Interferon‐Associated Inflammatory Program in BM‐MSCs

Having defined their foundational functional properties, we next sought to investigate the molecular pathways activated in BM‐MSCs upon LO exposure. To characterize the physical and molecular features of LOs, we first performed super‐resolution microscopy on LOs and sEVs isolated from bone metastatic prostate cancer PC3 cells. sEV identity was confirmed by positivity to a tetraspanin trio (CD9, CD63, and CD81) and LO identity by positivity to CD63 and RPN2 [10]. Individual LOs displayed micrometer‐scale dimensions, with particles >1 µm in diameter (Figure 2A,B and Figure S1A).

**FIGURE 2:**
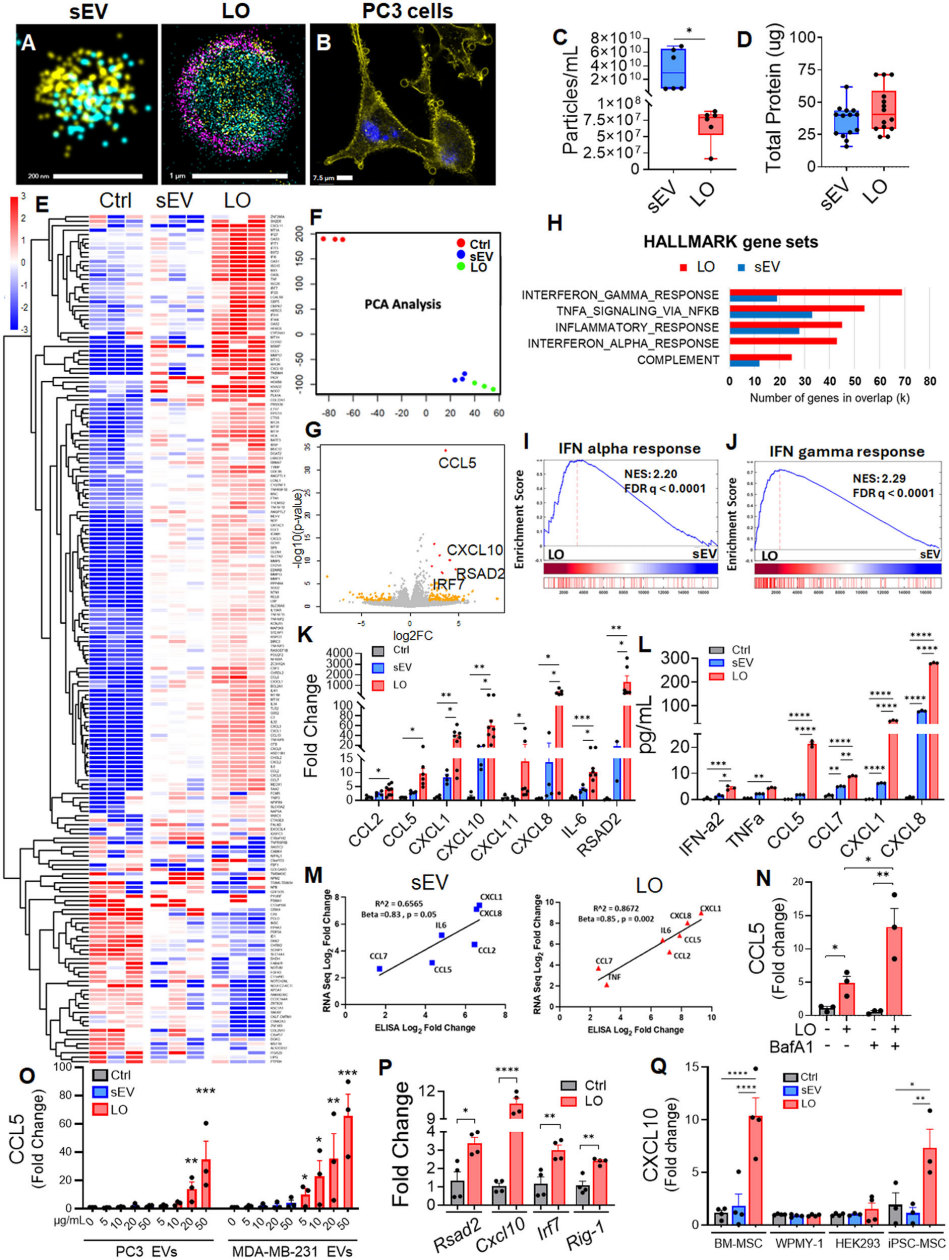
Large oncosomes (LOs) induce an interferon (IFN)‐associated inflammatory program in human bone marrow mesenchymal stem cells (BM‐MSC). (A) Super‐resolution direct Stochastic Optical Reconstruction super resolution microscopy (dSTORM) of PC3‐derived small extracellular vesicles (sEV) and LOs labeled with a pan‐EV membrane dye (cyan) and population‐specific markers. sEV were identified using tetraspanins cluster of differentiation (CD)9, CD63 and CD81 (yellow), whereas LOs were marked by CD63 (yellow) and ribophorin II (RPN2; magenta). Scale bar: 200 nm for sEV and 1 µm for LO. (B) Confocal microscopy imaging of PC3 cells stained with phalloidin‐rhodamine (yellow) and DAPI (blue) showing membrane blebbing. (C) Tunable‐resistive pulse sensing (TRPS) particle concentration measurements of PC3‐derived LO and sEVs. (D) Total protein (µg) abundance of PC3‐derived LO and sEV preparations. Each dot represents one independent experiment. (E) RNA sequencing (RNA‐seq) performed on human BM‐MSCs treated for 24 h with PC3‐derived LOs, sEV, or vehicle (PBS). Heatmap summarizing differentially expressed genes across RNA‐seq samples from LO‐, sEV‐, and vehicle‐treated human BM‐MSCs (*n* = 3 biological replicates per group). RNA‐seq differential expression analysis was performed using log2 fold‐change ranking with multiple‐testing correction as described in Section 4. (F) Principal component analysis (PCA) of RNA‐seq samples showing clustering by treatment condition. (G) Volcano plot showing differential gene expression in LO‐treated versus vehicle‐treated human BM‐MSCs. Points are colored according to log2 fold‐change and adjusted q‐value thresholds: Log2FC > 1 or < −1; *q* < 0.05. (H) Gene set enrichment analysis (GSEA) of MSigDB Hallmark gene sets (v7.0), showing the top enriched pathways in LO‐treated versus vehicle‐treated human BM‐MSCs, with corresponding overlap in sEV‐treated versus vehicle‐treated cells displayed for comparison. Bars indicate the number of overlapping genes. (**I, J)** GSEA enrichment plots for interferon alpha and gamma response in LO‐treated versus sEV‐treated BM‐MSCs. (K) Reverse transcription quantitative PCR (RT–qPCR) analysis of inflammatory gene expression in human BM‐MSCs treated with LO, sEV, or vehicle. Each dot represents one independent experiment. (L) Enzyme‐linked immunosorbent assay (ELISA) quantification of cytokines in supernatants from human BM‐MSCs treated with LO, sEV, or vehicle. (M) Correlation between RNA‐seq fold changes and cytokine protein levels measured by ELISA in human BM‐MSCs treated with sEV or LO. (N) RT–qPCR analysis of CCL5 expression in human BM‐MSCs treated with PC3‐derived LO following pre‐treatment with bafilomycin A1 (100 nM, 2 h). All RT–qPCR expression experiments were normalized to beta actin (ACTB). (O) RT–qPCR analysis of CCL5 expression in human BM‐MSCs treated with increasing concentrations of LO or sEV derived from PC3 or MDA‐MB‐231 cells. (P) RT–qPCR analysis of viperin (*Rsad2), Cxcl10, regulatory factor 7 (Irf7)*, and retinoic acid‐inducible gene I (*Rig‐I)* expression in mouse BM‐MSCs treated with 4T1.2‐derived LO. Expression was normalized to *ACTB*. (Q) CXCL10 expression analysis of BM‐MSC, WPMY‐1 (fibroblast), HEK293 (epithelial), and induced pluripotent stem cells (iPSC)‐MSC cells treated with either sEV or LO for 24 h. For all in vitro experiments, data represent at least three independent experiments with at least three biological and three technical replicates. Statistical analyses are described in Section 4.

Tunable resistive pulse sensing (TRPS) highlighted a modal distribution of LO diameter of 1744 nm in contrast with a modal distribution of sEV diameter of 120.8 nm (Figure 2C and Figure S1A). Transmission electron microscopy (TEM) confirmed the presence of intact, membrane‐enclosed vesicles in both the LO and sEV preparations (Figure S1B). Western blotting further demonstrated enrichment of the LO‐associated proteins heat shock protein 60 (HSP60) and heat shock protein family A member 5 (HSPA5 in LOs, whereas the canonical sEV markers tumor susceptibility gene 101 (TSG101), cluster of differetitation (CD) 9, and CD81 were enriched in sEVs, with no detectable signal for the tested cellular markers in either EV fraction (Figure S1C). Although sEVs were significantly more abundant than LOs, the total protein content was comparable between the two fractions (Figure 2C,D), indicating a higher protein amount per single LO than per single sEV. A similar pattern was observed in mouse‐derived bone metastatic 4T1.2 breast cancer cells (Figure S1D–H).

To define the transcriptional responses induced by EVs within the bone mesenchymal niche, RNA‐seq on primary human BM‐MSCs exposed to prostate cancer cell‐derived LOs or sEVs revealed substantially broader transcriptional reprogramming following LO treatment, including a large set of uniquely regulated transcripts (Figure 2E,F and Figure S2A,B).

LO‐treated BM‐MSCs displayed upregulation of inflammatory and interferon‐stimulated genes (ISGs), including CCL5, CXCL10, viperin (RSAD2), interferon regulatory factor 7 (IRF7), interferon‐stimulated gene 15 (ISG15), interferon‐induced protein with tetratricopeptide repeats (IFIT) family members, and interleucin 6 (IL6) (Figure 2E,G). Hallmark gene set enrichment analysis (GSEA) of the full ranked gene lists identified interferon gamma (IFNγ) response, tumor necrosis factor alpha (TNFα), nuclear factor kappa B signaling via NF‐κB, and inflammatory response among the programs induced by both EV populations. In contrast, the IFNα response was selectively enriched in LO‐treated cells and ranked among the most strongly upregulated Hallmark pathways only in the LO condition (Figure 2H–J).

To resolve how these transcriptional programs were distributed across distinct gene modules, we next performed unsupervised clustering of differentially expressed genes followed by cluster‐specific Hallmark GSEA correlation. Confirming the previous analysis, genes induced by both LOs and sEVs formed a shared inflammatory cluster. By contrast, genes preferentially induced by LOs formed a distinct cluster in which the IFNα response was the top enriched pathway (Figure S2C,D). Thus, although LOs and sEVs were derived from the same cancer cell model, they elicit partially overlapping inflammatory responses; in particular, LOs additionally induce a prominent type I interferon‐associated antiviral program, identifying them as distinct modulators of the bone marrow stromal niche.

Reverse transcription quantitative PCR (RT‐qPCR) confirmed the upregulation of interferon‐stimulated genes CCL2, CCL5, CXCL1, CXCL8, IL‐6, CXCL10, CXCL11, and RSAD2 in LO‐treated BM‐MSCs in comparison with ctrl and sEVs (Figure 2K). The changes in transcript abundance identified by RNA‐seq in BM‐MSCs were strongly reflected at the protein level in their conditioned medium (Figure 2L), with enzyme‐linked immunosorbent assay (ELISA) measurements showing a high degree of concordance with the corresponding gene expression changes (Figure 2M). LO treatment significantly induced CCL5 expression in BM‐MSCs, an induction that was markedly enhanced by Bafilomycin A1. Because Bafilomycin A1 alone did not increase CCL5 expression, these data indicate that inhibition of endosomal acidification and lysosomal degradation potentiate LO‐mediated inflammatory activation. This suggests that endolysosomal processing may regulate the availability of LO‐associated cargo for innate immune sensing in BM‐MSCs (Figure 2N).

Further experiments revealed that this response is dose‐dependent for LOs derived from prostate cancer PC3‐ and breast cancer MDA‐MB‐231‐derived cells (Figure 2O and Figure S2G,H). The response is observed not only in human but in murine recipient systems as well, since murine 4T1.2‐derived LOs similarly induced ISGs such as RSAD2, CXCL10, IRF7, and retinoic acid‐inducible gene I (RIG‐I) in murine BM‐MSCs (Figure 2P). LO treatment induced a significant increase in CXCL10 expression in BM‐MSCs. In contrast, WPMY‐1 and HEK293 cells remained largely unresponsive to LO exposure, suggesting that the LO‐induced response might not be a universal response across cell types. Notably, induced pluripotent stem cell (iPSC)‐derived MSCs also exhibited a significant response to LOs, supporting the idea that mesenchymal cells in the stroma are particularly sensitive to LO‐mediated inflammatory reprogramming (Figure 2Q). However, we cannot rule out the potential effect on other stromal, endothelial, and hematopoietic populations. Together, these findings identify a dose‐dependent, interferon‐associated inflammatory program induced by LOs in MSCs.

### 2.3 LO‐Mediated Inflammatory Reprogramming is Driven by Convergent Cytosolic DNA‐ and RNA‐Sensing Pathways

To identify the upstream pathways underlying LO‐induced inflammatory response in BM‐MSCs, we focused on transcripts selectively upregulated by LOs, which formed a distinct expression profile (Figure 3A), enriched for cytosolic nucleic acid sensing and STING‐mediated antiviral pathways (Figure 3B). The result was confirmed by Kyoto Encyclopedia of Genes and Genomes (KEGG) analysis, which revealed a specific LO‐induced enrichment of a cytosolic DNA‐sensing pathway (Figure 3C), in line with the presence of both internal and external DNA in LOs, validated using direct stochastic optical reconstruction super‐resolution microscopy (dSTORM) (Figure 3D).

**FIGURE 3:**
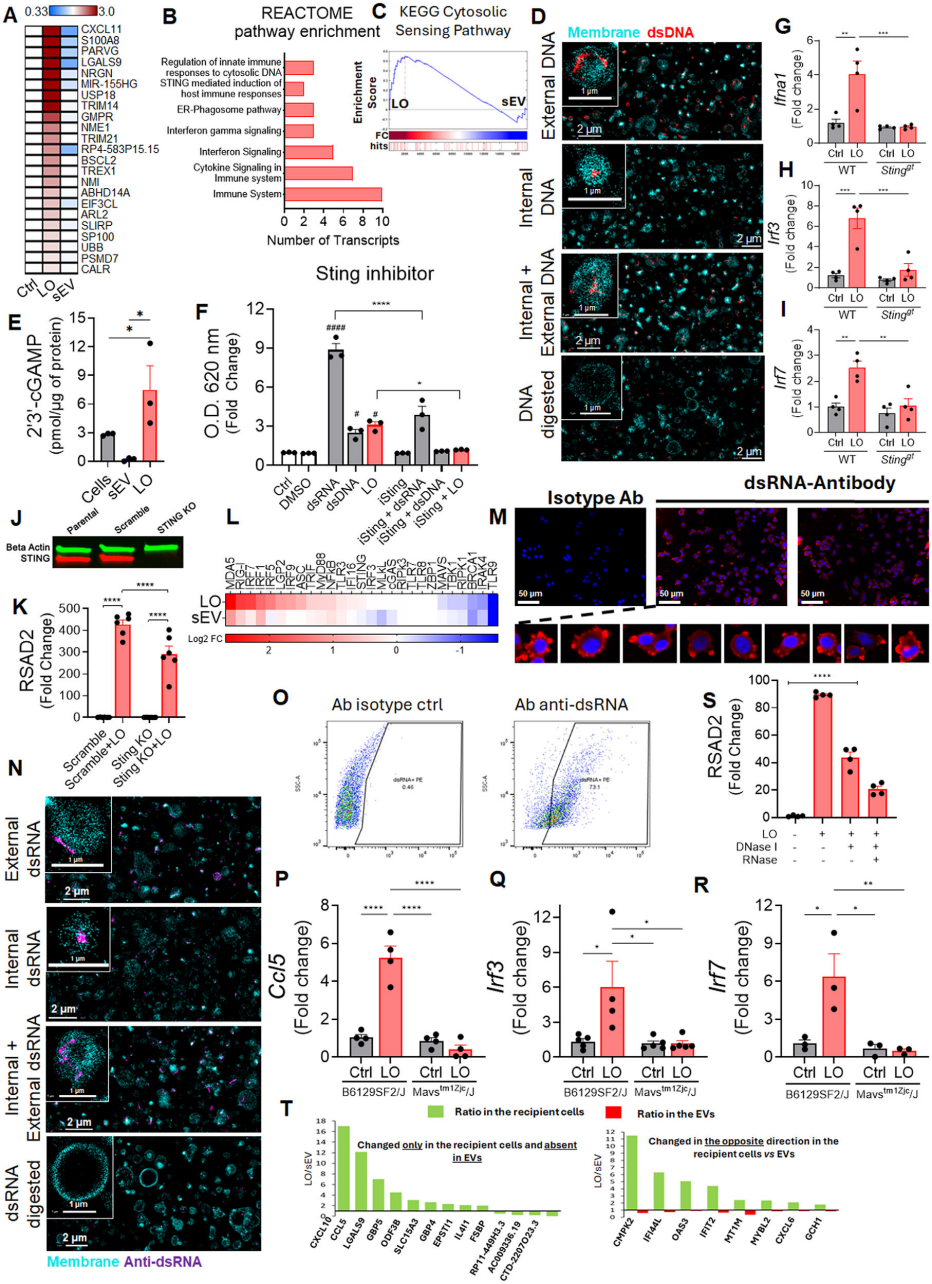
Large oncosomes (LOs) engage convergent cytosolic DNA‐ and RNA‐sensing pathways in recipient stromal cells. (A) Heatmap of transcripts uniquely upregulated, Log2 fold change of differentially expressed, and incongruent genes between LO‐treated and small extracellular vesicles (sEV)‐treated human bone marrow‐derived mesenchymal stem cells (BM‐MSCs) relative to vehicle (adjusted *p* < 0.05). (B) Reactome pathway enrichment analysis of the gene set shown in (A). (C) Gene set enrichment analysis (GSEA) of the Kyoto Encyclopedia of Genes and Genomes (KEGG) cytosolic nucleic acid–sensing pathway in LO‐treated versus sEV‐treated human BM‐MSCs. (D) Direct stochastic optical reconstruction super resolution microscopy (dSTORM) imaging of PC3‐derived LOs labeled with a pan‐EV membrane dye (cyan) and anti‐double stranded DNA (dsDNA) antibody (red). LO‐associated DNA was assessed under four conditions: no permeabilization (External DNA), permeabilization only (internal + external DNA), DNase + permeabilization (DNA digested), and sequential DNase digestion followed by permeabilization (internal DNA). DNase treatment for Internal DNA was performed prior to permeabilization to selectively remove exposed (external) DNA while preserving membrane‐protected internal DNA. Scale bars, 1 and 2 µm. (E) Competitive enzyme‐linked immunosorbent assay (ELISA) quantification of 2'3'‐cyclic GMP‐AMP (2′,3′‐cGAMP) in PC3 cell lysates, PC3‐derived sEV, and LO. (F) Secretable alkaline phosphatase (SEAP) reporter activity in Interferon Stimulated Genes (ISG) reporter cells treated with LO in the presence or absence of stimulator of interferon genes (STING) inhibitor. Poly(I:C) and poly(dA:dT) were included as positive controls. (G–I) Reverse transcription quantitative PCR (RT–qPCR) analysis of interferon regulatory factor 3 (*Irf3*) and 7 (*Irf7*)(G‐H),, and interferon alpha (*Ifna1)interferon* (I) expression in mouse bone marrow cells isolated from wild‐type or *Sting^Gt^* mice following LO treatment for 24 h. Expression was normalized to beta actin (*Actb)*. (J) Fluorescent western blot confirming STING knockout in human primary BM‐MSCs generated using CRISPR–Cas9. (K) RT–qPCR analysis of viperin (*RSAD2)* expression in STING‐knockout and scramble‐control human BM‐MSCs following LO treatment for 24 h. Expression was normalized to Actb. (L) Heatmap showing expression of nucleic acid‐sensing pathway genes in human BM‐MSCs treated with LO or sEV relative to vehicle, based on RNA‐seq data. (M) Immunofluorescence imaging of PC3 cells stained with anti‐double stranded RNA (dsRNA) antibody (red) or isotype control and counterstained with DAPI. Representative images and higher‐magnification views are shown. (N) dSTORM super‐resolution imaging of PC3‐derived LO labeled with a pan‐EV membrane dye (cyan) and anti‐dsRNA antibody (magenta). LO‐associated dsRNA was assessed under four conditions: no permeabilization (External dsRNA), permeabilization only (internal + external dsRNA), RNase III + permeabilization (dsRNA digested), and sequential RNase III digestion followed by permeabilization (internal dsRNA). RNase III treatment for Internal dsRNA was performed prior to permeabilization to selectively remove exposed (external) dsRNA while preserving membrane‐protected internal dsRNA. Scale bars, 1 and 2 µm. (O) Flow cytometry analysis of PC3‐derived LO (> 1 µm) stained with anti‐dsRNA antibody or isotype control. (P–R) RT–qPCR analysis of Ccl5 (P), Irf3 (Q), and Irf7 (R) expression in bone marrow cells isolated from B6;129SF2/J and B6;129‐Mavs^tm1Zjc^/J (Mavs‐deficient) mice and treated in vitro with PC3‐derived LO. (S) RT–qPCR analysis of *RSAD2* expression in human BM‐MSCs treated with LO pre‐incubated with DNase I or RNases. (T) Comparative analysis of gene expression changes in recipient human BM‐MSCs and corresponding EV preparations. Data are shown as LO/sEV ratios. Green bars indicate expression changes in recipient cells, whereas red bars indicate expression levels in EVs. Left, genes altered only in recipient cells and absent or negligible in EV cargo; right, genes showing opposite‐direction changes in recipient cells versus EVs, where response to sEV < response to LO in recipient cells but expression in sEV > expression in LO in EVs. For all in vitro experiments, data represent at least three independent experiments with at least three biological and three technical replicates. Statistical analyses are described in Section 4.

We have previously reported that LOs contain diverse DNA and RNA species [10, 16, 17, 18, 25, 26] that could engage recipient innate immune pathways, which might trigger cyclic GMP‐AMP synthase (cGAS) ‐ stimulator of interferon genes (STING) activation. Moreover, in comparison to sEVs and even to the donor cancer cells themselves, LOs contained higher levels of 2'3'‐cyclic GMP‐AMP (2’,3’‐cGAMP), the endogenous second messenger produced by cGAS upon DNA sensing (Figure 3E). This result suggests not only active shedding of this natural STING ligand in LOs, but also that LO‐associated 2’,3’‐cGAMP is a candidate contributor to recipient STING activation. Intriguingly, even if sEVs were used in a 1000‐fold excess in comparison to LOs, they did not contain detectable amounts of this molecule (Figure 3E).

Using an ISG reporter system stably lentivirally transduced into BM‐MSC, we found that LOs robustly activated reporter signaling at levels comparable to poly(dA:dT), a synthetic double‐stranded DNA used as a positive control (Figure 3F and Figure S3A) in recipient cells. A potent covalent small‐molecule inhibitor of STING (H151) abrogated both LO‐ and poly(dA:dT)‐induced activation of the reporter activity, corroborating the hypothesis that STING is associated with the interferon response induced by LOs (Figure 3F). Interestingly, STING inhibition also significantly reduced reporter activity in cells treated with the synthetic double‐stranded RNA poly(I:C), suggesting a potential involvement of STING not only in dsDNA but also in dsRNA‐associated signaling pathways.

To assess the contribution of STING signaling to LO‐induced inflammatory activation, we first treated mixed primary mouse bone marrow cultures, which included BM‐MSCs among other bone marrow cell populations, with LOs. LO treatment induced the expression of the antiviral genes Irf3, Irf7, and Ifna1 in wild‐type cultures, whereas this response was markedly impaired in bone marrow cells from mice lacking functional STING (Sting1^Gt/Gt^ mice) (Figure 3G–I). We next tested this pathway in human primary BM‐MSCs by generating CRISPR‐Cas9‐mediated knockout (KO) of the human STING1 gene (Figure 3J,K). Loss of STING attenuated, but did not completely abolish, LO‐induced RSAD2 expression. These results indicate that STING signaling contributes to the inflammatory response triggered by LOs, but also suggest that additional nucleic acid‐sensing pathways participate in this response, particularly in human BM‐MSCs.

To further investigate this possibility, we next examined the transcriptional landscape of nucleic acid‐sensing pathways induced by LOs. Further analysis of the transcriptional changes in response to PC3‐derived EVs confirmed a robust induction of multiple cytosolic RNA sensors, including melanoma differentiation‐associated protein 5 (*MDA5), RIG‐I*, and DExH‐box helicase 58 (DHX58/*LGP2)*, together with interferon regulatory factor family members such as *IRF1, IRF5, IRF7*, and *IRF9*, in LO‐treated BM‐MSCs (Figure 3L). GSEA further confirmed enrichment of a double stranded RNA (dsRNA) response in LO‐treated cells (Figure S3B).

Given these findings, we next investigated whether dsRNA was associated with LO biogenesis and cargo. PC3 cells stained with a dsRNA antibody exhibited dsRNA accumulation in correspondence with the plasma membrane blebs (Figure 3M), showing dsRNA signal associated with membrane blebs. In confirmation, dSTORM imaging of LOs revealed dsRNA association with LOs both internally and externally (Figure 3N). In parallel, flow cytometric characterization of LOs revealed that over 50% of particles larger than 1 µm stained positive for dsRNA, suggesting substantial accumulation of dsRNA within LOs (Figure 3O and Figure S3C).

To functionally evaluate the contribution of cytosolic RNA sensing to the stromal response induced by LOs, we next examined the mitochondrial antiviral‐signaling protein (Mavs) pathway, a central downstream signaling adaptor shared by both MDA5 and RIG‐I. In primary bone marrow cells obtained from B6;129SF2/J (wild‐type) and B6;129‐Mavs^1Zjc^/J (Mavs KO) mice, LO treatment induced *Ccl5, Irf3*, and *Irf7* in wild‐type but not Mavs KO BM cells (Figure 3P–R). Together, these results demonstrate that LO‐associated dsRNA activates cytosolic RNA‐sensing pathways in recipient cells and further support a model in which LOs engage redundant nucleic acid‐sensing mechanisms converging on inflammatory interferon signaling.

To directly assess the contribution of LO‐associated nucleic acids to this inflammatory response, we next permeabilized the LOs and enzymatically degraded DNA and RNA associated with them before treating the recipient cells. Degradation of LO‐associated nucleic acids attenuated LO‐induced inflammatory signaling, with DNase I treatment significantly reducing RSAD2 and CXCL8 expression in BM‐MSCs and combined DNA/RNA degradation resulting in an even greater suppression (Figure 3S and Figure S3D).

Finally, to determine whether LO‐induced transcriptional alterations were due to activation, in response to these EVs, of endogenous transcriptional programs or a mere reflection of the transfer of EV‐associated mRNAs to the recipient cells, we compared RNA abundance in LOs and sEVs with the transcriptional response induced by these two EV populations in BM‐MSCs. Transcripts more strongly upregulated by LOs in recipient cells were not necessarily enriched in LO themselves (Figure 3T). We then compared, for each transcript, the LO/sEV expression ratio in recipient BM‐MSCs with the corresponding LO/sEV ratio in the EV populations themselves. First, a substantial fraction of transcripts was altered exclusively in recipient cells while absent or negligible in EV cargo, consistent with endogenous transcriptional activation downstream of EV‐mediated signaling (Figure 3T, left). Second, a subset of genes displayed discordant behavior, being more strongly induced by LOs in recipient cells despite being more abundant in sEV cargo, arguing against direct RNA transfer as the primary driver of these responses (Figure 3T, right). Collectively, this supports a model in which LOs primarily induce inflammatory reprogramming of BM‐MSCs through activation of endogenous host transcriptional programs rather than through delivery of functional mRNA cargo.

### 2.4 LO‐Induced Stromal Reprogramming Promotes Neutrophil Recruitment and Phenotypic Changes

Having identified LO‐associated cargo and recipient innate immune responses (Figure 3), we next asked whether the response to LOs translates into functional changes in immune cell recruitment and phenotypic remodeling within the bone microenvironment. Transcriptomic analysis of LO‐treated BM‐MSCs revealed an upregulation of chemotactic genes. A heatmap of the top 50 upregulated genes revealed significant enrichment of transcripts associated with leukocyte trafficking and neutrophil migration in LO‐treated cells, a pattern that was largely absent in sEV‐treated cells (Figure 4A). Consistently, GSEA demonstrated strong enrichment of the gene ontology (GO) for neutrophil chemotaxis signature in LO‐treated BM‐MSCs vs ctrl (normalized enrichment score (NES) = 3.71) compared to sEVs vs ctrl (NES = 2.66; Figure 4B,C).

**FIGURE 4:**
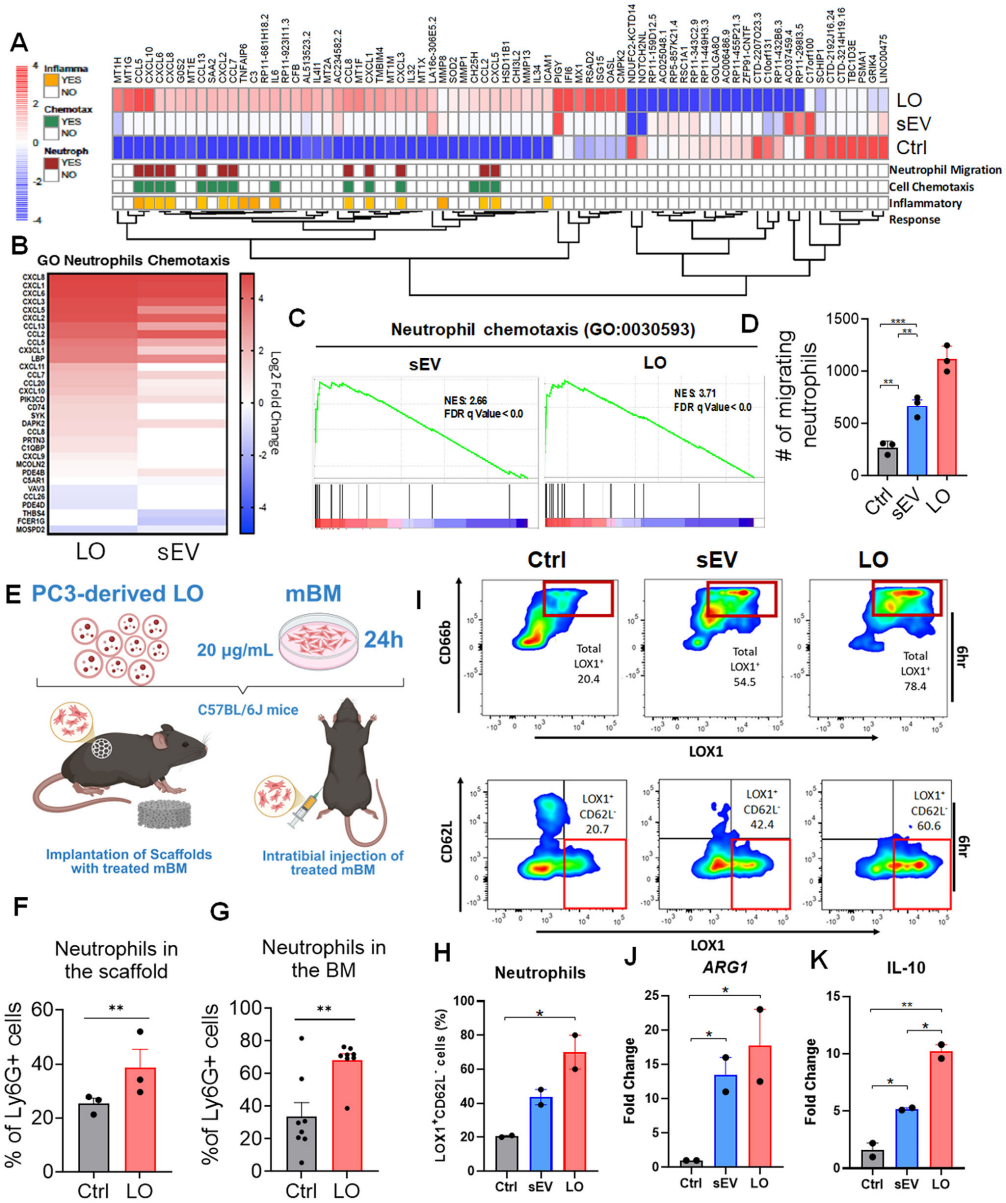
Large oncosome (LOs)‐induced reprogramming of bone marrow‐derived mesenchymal stem cells (BM‐MSCs) promotes recruitment and differentiation of neutrophils into polymorphonuclear myeloid‐derived supressor cells (PMN‐MDSC)‐like phenotype. (A) Heatmap of the top 50 upregulated genes in human BM‐MSCs treated with LO relative to vehicle control, based on the RNA‐seq dataset described in figure 2A (*n* = 3 biological replicates per group). (B, C) Gene set enrichment analysis (GSEA) of the Gene Ontology (GO) neutrophil chemotaxis pathway (GO:0030593) in LO‐treated or small extracellular vesicles (sEV)‐treated versus vehicle‐treated human BM‐MSCs, ranked by log2 fold change. (D) Transwell migration assay quantifying recruitment of primary human neutrophils freshly isolated from peripheral blood toward conditioned medium from vehicle‐, sEV‐, or LO‐treated human BM‐MSCs. Each dot represents one independent experiment (*n* = 3/group). (E) Experimental schematic of neutrophil infiltration into either subcutaneous scaffolds seeded with vehicle‐ or LO‐treated mouse bone marrow (mBM) and implanted subcutaneously or vehicle‐ or LO‐treated mBM injected intratibially (*n* = 4/group) into C57BL/6J mice. Scaffolds were harvested after 1 week (n = 3 mice per group) post implantation and bone marrows harvested 48 h after injection. (F–H) Quantification of the percentage of lymphocyte antigen 6 complex locus G (Ly6G)^+ cells^ from the total cluster of differentiation (CD) 45^+^ cells in the scaffold (F) and bone marrow (G). (H, I) Flow cytometry analysis of primary human neutrophils exposed for 6 h to conditioned medium from vehicle‐, sEV‐, or LO‐treated human BM‐MSCs, assessing CD62L, CD66b, and lectin‐type oxidized LDL receptor 1 (LOX‐1) expression. (J, K) Reverse transcription quantitative PCR (RT–qPCR) analysis of arginase 1 (ARG1; J) and interleucin 10 (IL10; K) expression in primary human neutrophils following 6 h exposure to conditioned medium from vehicle‐, sEV‐, or LO‐treated BM‐MSCs. Expression was normalized to beta ctin (ACTB). Each dot represents one independent experiment.

To assess the functional impact of this transcriptional remodeling, we performed transwell migration assays using conditioned medium from EV‐treated BM‐MSCs, confirming a significantly increased neutrophil migration in LO‐treated compared to the vehicle‐ or sEV‐treated cells conditioned medium (Figure 4D). To validate these observations in vivo, we employed two complementary approaches. LO‐educated C57BL/6J bone marrow cells were either embedded within 3D polyurethane scaffolds [39] and implanted subcutaneously into syngeneic mice (Figure 4E,F) or injected directly into the tibiae of syngeneic recipients (Figure 4E,G). In both cases, we observed a significant increase in lymphocyte antigen 6 complex locus G (Ly6G)^+^ neutrophils in response to the LO‐treated cells.

We next examined whether LOs might modulate the neutrophil phenotype. Human neutrophils exposed to conditioned medium from LO‐treated BM‐MSCs displayed a significant increase in Lectin‐type oxidized LDL receptor 1 (LOX‐1) and CD66b expression and near‐complete loss of CD62L. This result was time‐dependent and visible even after just 1 h of exposure to the conditioned medium (Figure 4H,I and Figure S4). LOX‐1+ neutrophils are a specialized immature subset of neutrophils that function as Polymorphonuclear Myeloid‐Derived Suppressor Cells (PMN‐MDSCs) that are known to heavily suppress T‐cell immune responses in cancer [40]. Upregulation of the immunosuppressive genes arginase 1 (ARG1) and interleucin 10 (IL10) (Figure 4J,K) further suggested a functional polarization toward a PMN‐MDSC‐like state.

### 2.5 Tumor‐Derived LO Shedding Contributes to Systemic PMN‐MDSC Accumulation and Tumor Progression

Building on our findings that LO‐induced programming of BM‐MSCs promotes neutrophil accumulation and PMN‐MDSC‐like polarization (Figure 4), we next asked whether tumor‐derived LOs are required to drive this systemic immunosuppressive neutrophil response in vivo, by developing an orthotopic breast cancer model with Septin 2 (Sept2) knockout to impair LO shedding (Figure 5A). Sept2 is a conserved GTP‐binding cytoskeletal protein implicated in membrane blebbing and cortical organization in cancer cells, including bleb‐associated signaling in detached melanoma cells [41], although its involvement in LO biogenesis has not been previously tested [42]. Efficient loss of Sept2 following CRISPR‐Cas9‐mediated editing was confirmed by immunoblotting across independent monoclonal 4T1.2 populations (Figure 5B). Notably, we observed a marked reduction in the release of large particles from Sept2‐deficient cells compared with scramble controls (Figure 5C), identifying Sept2 as a potential regulator of LO shedding. BALB/c mice were then implanted with either wildtype (WT) or Sept2^KO^ 4T1.2 cells, while additional matched cohorts received systemic LO injections as a rescue strategy (Figure 5A). Tumor size was comparable between groups, as assessed by caliper measurements (Figure S5A). dSTORM analysis of plasma‐derived EVs revealed a significant reduction in circulating phosphatidylinositol glycan anchor biosynthesis class S (PIGS)^+^ and Caveolin‐1 (CAV1)^+^ large particles in mice bearing Sept2 KO tumors, confirming the ability of Sept2 loss to suppress LO abundance in vivo.

**FIGURE 5:**
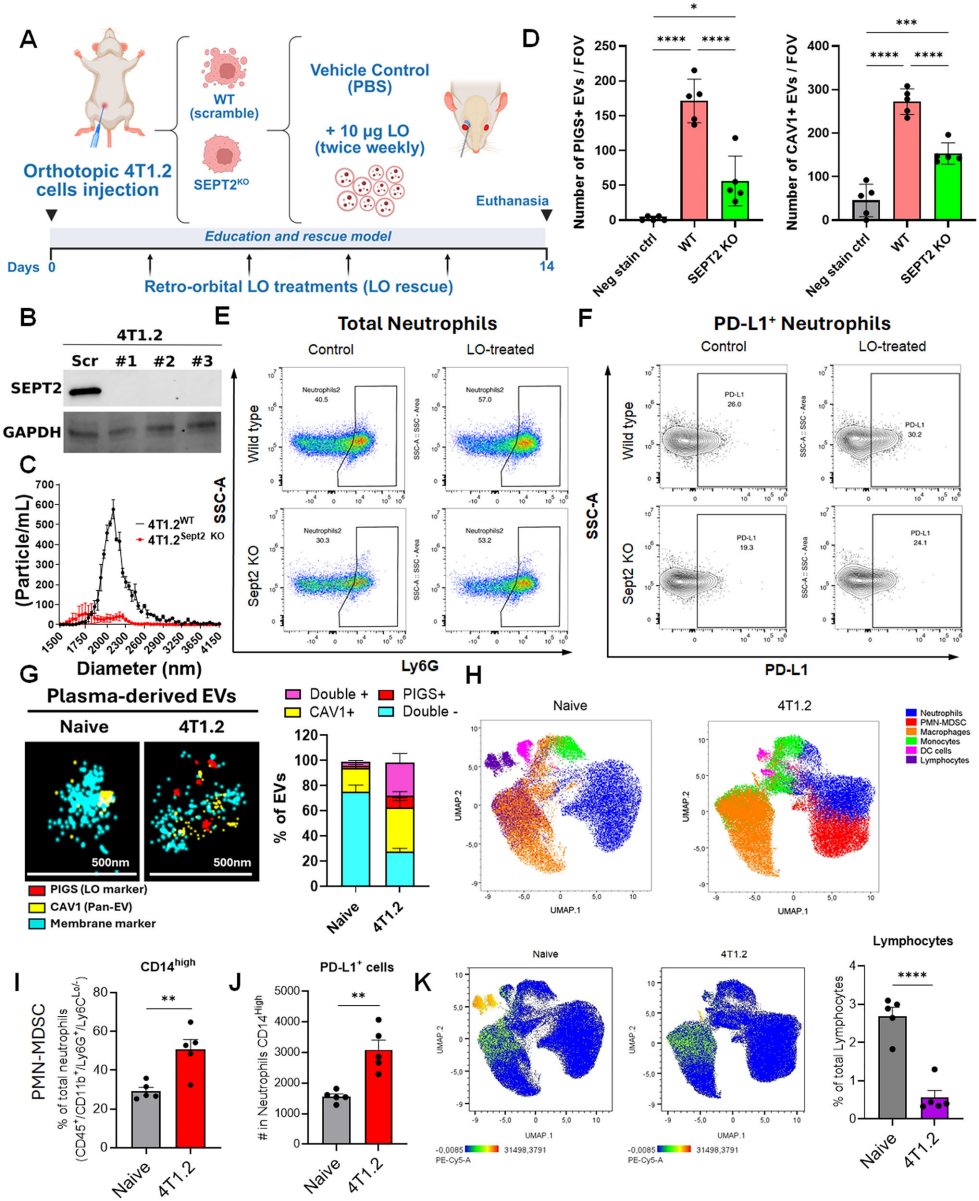
Tumor large oncosomes (LOs) shedding is associated with bone marrow neutrophil remodeling. (A) Schematic representation of the LO‐deficient and LO‐rescue orthotopic tumor model developed. Balb/c mice were orthotopically injected into the mammary fat pad with 4T1.2 cells engineered to lack septin 2 (Sept2) (LO‐deficient) or not. A separate experimental group was set up, including systemic injection of LO to rescue LO‐induced signaling in the absence of Sept2. (B) SEPT2 western blot analysis of scramble control and 3 individual monoclonal populations of 4T1.2 Sept2 knockout induced using CRISPR‐Cas9. (C) Microfluidic resistive pulse sensing particle count analysis quantifying LO shedding in scramble control and Sept2 KO 4T1.2 cell lines. (D) Quantification of the total number of particles per field of view (FOV) positive for phosphatidylinositol glycan anchor biosynthesis class S (PIGS) (LO marker) and Caveolin 1 (CAV1) (Pan‐EV marker) in the plasma from the tumor‐bearing mice described in figure 5A. (E, F) Spectral flow cytometry analysis of the bone marrow of the mice in figure 5A, showing the percentage of lymphocyte antigen 6 complex locus G (Ly6G)^+^ cells (neutrophils) (E) and the percentage of programmed death‐ligand 1 (PD‐L1)^+^ neutrophils (polymorphonuclear myeloid‐derived supressor cells (PMN‐MDSC)) (F). (G) Direct stochastic optical reconstruction super resolution microscopy (dSTORM) analyses of particles isolated from the plasma of BALB/c mice naïve vs orthotopically implanted with the bone metastatic 4T1.2 breast cancer cell line with high LO shedding. Imaging (left) and quantification (right) of the percentage of particles positive or negative for either PIGS (red) or CAV1 (yellow). A general membrane dye was used for counterstaining (cyan). (H) Uniform manifold approximation and projection (UMAP) visualization and flow cytometry data analysis using self‐organizing maps (FlowSOM) clustering of high‐parameter spectral flow cytometry data from bone marrow cells of naïve and 4T1.2 tumor‐bearing mice. Data represent concatenated files from all animals (*n* = 5 mice per group). (I) Quantification of PMN‐MDSC in the flow cytometry analysis from panel 6H. (J) PD‐L1 expression within the PMN‐MDSC cluster identified in panel 6I. (K) UMAP visualization (left) and quantification (right) of cluster of differentiation 3 (CD3) expression across bone marrow immune populations from naïve and tumor‐bearing mice. Statistical analyses are described in Section 4.

We next investigated how tumor‐derived LO production shapes the composition of bone marrow myeloid populations. Neutrophils and PMN‐MDSCs were identified using a sequential gating strategy based on CD45, CD11b, Ly6G, and programmed death‐ligand 1 (PD‐L1) expression (Figure S5C). Spectral flow cytometry revealed a reduction in the overall frequency of Ly6G^+^ neutrophils in mice bearing Sept*2* KO tumors relative to controls (Figure 5E). More strikingly, the proportion of PD‐L1^+^ neutrophils, consistent with a PMN‐MDSC phenotype, was significantly decreased in the absence of tumor‐derived LOs (Figure 5F). Importantly, in an LO‐rescue model, systemic administration of purified syngeneic LOs restored PD‐L1^+^ neutrophil accumulation within the BM compartment, suggesting that LOs are indeed sufficient to drive this immunosuppressive phenotype in vivo (Figure 5F).

To investigate how prolonged tumor progression influences the BM compartment, we compared healthy and tumor‐bearing mice using the highly metastatic, LO‐producing 4T1.2 orthotopic breast cancer model. BALB/c mice were monitored for four weeks following mammary fat pad implantation, after which bone marrow was collected for flow cytometric analysis (Figure S5B). Super‐resolution dSTORM imaging confirmed a substantial increase in the proportion of circulating PIGS^+^ large particles in mice bearing orthotopic 4T1.2 tumors compared to naïve controls (Figure 5G). High‐dimensional spectral flow cytometry, combined with uniform manifold approximation and projection (UMAP) and flow cytometry data analysis using self‐organizing maps (FlowSOM) clustering, revealed a significant expansion of neutrophil populations within the BM of tumor‐bearing animals (Figure 5H). More specifically, we observed a significant increase in the frequency of cells with high expression of CD14, CXCR2, and PD‐L1, and reduced expression of major histocompatibility complex class II (MHC‐II) (Figure 5I,J and Figure S5C–E), compatible with a PMN‐MDSC phenotype.

Notably, PMN‐MDSC abundance in the BM positively correlated with primary tumor size, indicating a link between tumor growth and BM expansion of this immunosuppressive population (Figure S5F). BM cells further exhibited a marked reduction in CD3^+^ lymphoid populations (Figure 5K), consistent with the establishment of an immunosuppressive niche. These observations occurred in the absence of detectable GFP‐positive tumor cells in the bone marrow, suggesting that LO‐associated immunological remodeling may precede detectable metastatic colonization. However, rare disseminated tumor cells below the assay detection limit cannot be excluded, and establishing this temporal sequence will require controlled experiments separating LO exposure from tumor‐cell inoculation, together with serial bone marrow analyses using more sensitive tumor‐cell detection methods. Notably, no significant changes were observed in macrophage populations (Figure S5G,H), indicating that the LO‐driven remodeling identified in this study, which results in the upregulation of cytokines known to regulate myeloid cells, preferentially impacts the neutrophil compartment in vivo.

Together, these data identify a previously unrecognized mechanism by which tumor‐derived LOs shape the metastatic niche. By reprogramming bone marrow stromal cells toward a pro‐inflammatory, neutrophil‐accumulating state, LOs drive the polarization of immunosuppressive PMNs while promoting lymphocyte depletion. These findings, collectively, establish LOs as systemic mediators of metastatic niche formation and provide a mechanistic link between tumor‐derived vesiculation, stromal reprogramming, and early immune remodeling at distant sites.

## 3 Discussion

LOs have emerged as a distinctive class of tumor‐derived EVs associated with metastatic and lethal cancer [12, 13], yet their functional role in shaping metastatic progression has remained incompletely defined. In this study, we provide evidence that LOs act as mediators of inflammatory reprogramming in bone marrow stromal cells, associated with neutrophil remodeling and metastatic colonization. By integrating cargo characterization, transcriptomic profiling, and in vivo functional models, we establish LOs as specialized and highly complex signaling platforms that orchestrate inflammatory and immunosuppressive remodeling of the bone microenvironment.

Mechanistically, this study identifies cytosolic nucleic acid sensing as the central pathway underlying LO‐induced stromal activation. LOs have emerged as the EV population enriched in nucleic acids [13, 17, 18, 22, 26]. In our findings, this translates into an unexplored mechanism of intercellular communication where distinct nucleic acid species converge on redundant signaling pathways, specifically STING for DNA and MAVS (MDA5/RIG‐I) for dsRNA, within recipient cells. The convergence of DNA and RNA sensing pathways suggests that LOs act as composite carriers of immunostimulatory signals capable of activating a coordinated innate immune response. Consistent with this concept, recent work has shown that epigenetic reprogramming through enhancer of zeste homolog 2 (EZH2) inhibition induces accumulation of endogenous dsRNA and activates a dsRNA‐STING‐interferon signaling axis in prostate cancer models [43].

In addition, a particularly intriguing observation is the high enrichment of 2’3’‐cGAMP within LOs. As a direct activator of STING, 2’3’‐cGAMP could allow LOs to propagate innate immune signaling constitutively and independently of upstream DNA sensing in recipient cells, thereby amplifying and accelerating immune activation in distant compartments. This suggests a potential mechanistic basis by which LO‐derived nucleic acids may elicit stronger interferon responses than those delivered by sEVs. Such a mechanism may be especially relevant in the context of metastatic dissemination, where reprogramming of the microenvironment is required to support tumor cell colonization [44]. However, the hypothesis that detection of 2’3’‐cGAMP in LO preparations identifies a candidate route for recipient STING activation still requires direct functional testing.

Findings from this study also define a stromal‐mediated axis through which tumor‐derived vesicles indirectly shape immune composition, linking vesicle signaling to the expansion of immunosuppressive myeloid populations. PMN‐MDSCs are increasingly recognized as central mediators of metastatic immune remodeling, but the upstream tumor‐derived signals that promote their accumulation in distant organs remain incompletely defined. Our findings support a model in which LOs act as systemic messengers that reprogram the bone marrow microenvironment before metastatic expansion. Across complementary in vitro and in vivo approaches, LO exposure was associated with activation of a pro‐inflammatory stromal program, increased production of neutrophil‐ and myeloid‐recruiting mediators, accumulation and polarization of immunosuppressive PMN‐MDSC populations, and concomitant depletion of lymphoid cells in the bone marrow. These observations suggest that LOs do not merely promote tumor cell‐intrinsic metastatic traits but also remodel distant stromal and immune compartments to support a metastasis‐permissive marrow environment.

Interestingly, a recent study demonstrated that mitochondrial DNA released by senescent tumor cells enhances PMN‐MDSC‐driven immunosuppression through activation of the cGAS‐STING pathway [31]. This mechanism may plausibly be mediated by LOs, as we have previously reported using multi‐omics profiling that LOs are enriched in mitochondrial content (e.g., mitochondrially encoded cytochrome c oxidase I (MT‐CO1) and translocase of outer mitochondrial membrane 40 (TOMM40)) [10], suggesting a potential route for the transfer of immunostimulatory mitochondrial DNA to recipient cells. These discoveries extend prior models of “seed and soil” crosstalk. While previous work linked LOs to local prostate fibroblast reprogramming via AKT serine/threonine kinase 1 (AKT)‐MYC proto‐oncogene (MYC) [21], this work uncovers a long‐range axis targeting the stroma at the most frequent metastatic site for both prostate and breast cancer.

Our findings also provide important context for the role of IFN signaling in cancer. While IFN pathways are classically associated with anti‐tumor immunity, accumulating evidence suggests that chronic or spatially restricted activation can promote immunosuppressive and pro‐tumorigenic effects [45, 46]. The sustained IFN response induced by LOs in stromal cells appears to drive chemokine production and myeloid cell recruitment in a manner that ultimately favors tumor progression. This highlights the context‐dependent nature of innate immune signaling and suggests that tumor‐derived vesicles may exploit these pathways to reprogram the host environment.

However, metastatic colonization can also be shaped by tumor‐cell‐intrinsic programs that can support bone metastasis without experimentally imposed EV preconditioning [47, 48, 49]. Within this broader framework, our findings support LO‐associated stromal remodeling as a contributor to metastatic progression in the models examined, without establishing it as a universal prerequisite for colonization.

From a translational perspective, our findings suggest several directions for future investigation. Studies targeting LO biogenesis, release, or uptake could determine whether disrupting this communication reduces stromal conditioning and metastatic progression. In parallel, LO‐associated surface markers and nucleic acid cargo [10, 17] could be evaluated as candidate biomarkers of tumor‐associated stromal remodeling and as molecular features that may enable selective targeting. Exploring approaches to intercept circulating LOs would provide an opportunity to test whether limiting their access to distant tissues alters niche formation or subsequent metastatic outgrowth. These investigations should consider both early conditioning and ongoing niche remodeling, given the intrinsic capacity of tumor cells to colonize distant organs and the clinically occult dissemination that may already be present at diagnosis. If efficacy, specificity, and safety are established, LO‐directed approaches could subsequently be evaluated alongside treatments targeting primary tumors or established metastases, including studies testing whether modulation of the stromal and immune environment influences responses to immunotherapy. Advancing these possibilities will require validation of LO‐associated functional activity in patient samples, assessment of its relationship to myeloid phenotypes and disease progression, and evidence that LO‐mediated signaling represents a consequential and therapeutically tractable component of human metastatic disease.

Our data support a role for LO‐associated nucleic acids in stromal activation, with further investigation needed to resolve the individual contributions of DNA, RNA, and cGAMP and the mechanisms governing LO uptake, intracellular trafficking, and functional cargo delivery. BM‐MSCs are a responsive recipient population, but experiments using mixed marrow cultures do not identify the cellular origin of all responses, and the contributions of other marrow populations in vivo remain to be defined. The observed neutrophil phenotypes also warrant direct functional assessment of immunosuppressive activity.

The broader relevance of these findings requires consideration of the experimental models and exposure conditions. The in vivo metastatic studies rely primarily on the immunocompetent 4T1.2 breast cancer model, leaving generalizability to other tumor types and immune contexts unresolved. Protein‐normalized EV inputs and systemic bolus administration do not establish equivalent particle or bioactive cargo doses or fully reproduce endogenous exposure, limiting conclusions about the relative contributions of LOs and sEVs to metastatic progression. Sept2 perturbation and LO rescue provide complementary evidence linking LO shedding to marrow immune remodeling, although additional effects of Sept2 loss on tumor‐cell properties or other secreted components cannot be excluded. Furthermore, while MSC priming preceded tumor challenge, studies in tumor‐bearing mice cannot exclude rare, disseminated cells below assay detection limits; the observed remodeling may therefore encompass both pre‐metastatic conditioning and subsequent niche evolution. Establishing clinical relevance will require validation of patient‐derived LO activity and its relationship to myeloid phenotypes and disease progression.

In summary, we propose a model in which tumor‐derived LOs function as systemic signaling hubs that deliver immunostimulatory nucleic acids and second messengers to stromal cells, activating innate immune pathways and driving the formation of an inflammatory environment which, through the acquisition of an immunosuppressive polarization counterbalance, can culminate in the formation of a tumor‐supportive metastatic niche.

Through this mechanism, LOs link tumor cell plasticity and vesiculation to long‐range immune remodeling, thereby facilitating metastatic progression. These findings redefine the functional landscape of tumor‐derived EVs and position LOs as potential mediators of tumor‐host communication in cancer metastasis.

## 4 Materials and Methods

### 4.1 Cell Culture and Primary Cell Preparation

The human prostate cancer cell line PC3 and human breast cancer cell line MDA‐MB‐231 were maintained in Dulbecco's Modified Eagle Medium (DMEM; GIBCO). The murine breast cancer cell line 4T1.2 was maintained in alpha Minimum Essential Medium (alpha‐MEM; GIBCO). Human primary bone marrow‐derived mesenchymal stem cells (BM‐MSCs) (ATCC) were cultured in MesenPRO RS medium (GIBCO) according to the manufacturer's recommendations. Murine bone marrow cells and BALB/c‐derived OriCell mouse BM‐MSCs (Cyagen) were maintained in OriCell Complete Medium for Mouse BM‐MSCs (Cyagen). Unless otherwise indicated, media were supplemented with 10% fetal bovine serum, 2 mM L‐glutamine, and 1% penicillin–streptomycin. Cells were cultured at 37°C in a humidified incubator with 5% CO_2_. Primary murine bone marrow cells were isolated from long bones of WT B6;129SF2/J mice and genetically modified strains including B6;129‐Mavs^1Zjc^/J and Sting1^gt^ mice. Femurs and tibiae were aseptically harvested, cleaned of adherent tissue, and flushed with pre‐warmed culture medium using a sterile syringe. Cell suspensions were filtered through a 70 µm cell strainer, red blood cells were lysed using ammonium–chloride–potassium (ACK) lysis buffer, and cells were washed and resuspended in the appropriate culture medium. Primary human neutrophils were isolated from freshly collected peripheral blood from healthy donors using the EasySep Direct Human Neutrophil Isolation Kit according to the manufacturer's protocol and maintained in RPMI‐1640 medium. Human primary neutrophil‐related studies were conducted under Cedars‐Sinai Institutional Review Board approval IRB 19627 and in accordance with institutional and ethical guidelines. Written informed consent was obtained from all participants prior to the collection and use of human samples in this study. All human and murine cell lines were obtained from ATCC unless otherwise indicated, and primary BM‐MSCs were obtained commercially and used at low passage. Cell lines were used at a maximum passage of 12, and primary cells were used at passage ≤ 6. Cultures were routinely monitored for mycoplasma contamination using MycoStrip Mycoplasma Detection Kit (InvivoGen) and/or MycoAlert PLUS (Lonza). Cultures used for EV production had at least 98% viability before conditioned medium collection, as assessed by trypan blue exclusion or equivalent viability testing.

### 4.2 Mouse Models

All animal experiments were approved by the Cedars‐Sinai Institutional Animal Care and Use Committee under protocol IACUC008508 (PI: Di Vizio) and were performed in accordance with institutional guidelines for animal care and use. Mice were purchased from The Jackson Laboratory and used between 6 and 8 weeks of age. Male mice were used for prostate cancer and female mice for breast cancer, as appropriate for each experimental model. BALB/cJ mice were used for syngeneic orthotopic 4T1.2 breast cancer models, intratibial BM‐MSC priming experiments, and LO rescue studies. C57BL/6J mice were used for bone marrow stromal and immune recruitment assays when indicated. WT B6;129SF2/J mice, Sting1^gt^ mice, and B6;129‐Mavs^1Zjc^/J mice were used for genetic validation of cytosolic nucleic acid‐sensing pathways in primary bone marrow cultures. Animals were housed under standard pathogen‐free conditions with ad libitum access to food and water and were monitored routinely according to approved veterinary and IACUC guidelines.

### 4.3 In Vivo BM‐MSC Priming and Intracardiac Breast Cancer Metastasis Model

BALB/c‐derived mouse BM‐MSCs (Cyagen OriCell) were pretreated with nLuc‐GFP 4T1.2‐derived LOs, sEVs, or vehicle control and injected into the tibia of syngeneic female BALB/c mice. A total of 1 × 10^5^ BM‐MSCs in 10 µL were injected per tibia. Forty‐eight hours later, 5 × 10^4^ GFP/NanoLuc reporter 4T1.2 cells in 100 µL PBS were injected into the left cardiac ventricle. Mice were monitored for 2 weeks by IVIS imaging using Nano‐Glo Fluorofurimazine In vivo Substrate Kit (Promega) to quantify metastatic spread and focal tibial lesions. All mice were female and 6–8 weeks old.

### 4.4 Orthotopic 4T1.2 Tumor Education and Breast Cancer Metastasis Model

For systemic EV education during primary tumor progression, female 6–8‐week‐old BALB/c mice were orthotopically implanted with 2 × 10^5^ nanoLuc‐GFP 4T1.2 cells into the lower right mammary fat pad in Corning Matrigel growth factor‐reduced basement membrane matrix, phenol red‐free. Mice were assigned to vehicle, sEV, or LO education groups (15 animals per group). During the 14‐day primary tumor growth phase, mice received four retro‐orbital EV injections split across the 2‐week interval, each consisting of 10 µg EV protein in 100 µL PBS. Primary tumors were surgically excised on day 14. Metastatic progression was monitored for an additional 60 days, and mice were euthanized at approximately day 75 or earlier if humane endpoints were reached. Primary tumor growth was measured during the initial phase, and metastatic burden was tracked by IVIS using Nano‐Glo Fluorofurimazine In vivo Substrate Kit (Promega) and lesion analysis.

### 4.5 Hollow Fiber Bioreactor Culture

For large‐scale EV production, selected cancer cell lines (PC3 and 4T1.2) were expanded in a hollow fiber bioreactor using C2018 cartridges (FiberCell Systems) with a 20 kDa molecular weight cutoff. Cells were seeded into the extracapillary space without prior coating at an initial target input of approximately 1 × 10^9^ cells. Cultures were maintained under continuous perfusion in high‐glucose DMEM, and fetal bovine serum was gradually replaced with CDM‐HD according to the manufacturer's recommendations, until reaching a serum‐free condition and 10% CDM‐HD. Bioreactor cultures were monitored by glucose consumption and maintained under high‐density conditions, with glucose uptake targeted at approximately 1–1.5 g/day. Extracapillary space medium was collected every other day for EV enrichment. Bioreactors were maintained for up to 60 days, with culture health monitored by glucose consumption, absence of overt cell contamination/dead cells in conditioned medium, and confirmation of EV markers after isolation. Cultures were routinely monitored for mycoplasma.

### 4.6 Enrichment and Purification of LOs and sEVs From Conditioned Medium

PC3 and MDA‐MB‐231 cell lines were selected as highly blebbing human prostate and breast cancer models with robust LO production. EV enrichment was performed from conditioned medium collected from conventional 2D cultures or hollow fiber bioreactor cultures, following an adapted differential centrifugation and iodixanol density‐gradient purification workflow. For 2D culture, cells were plated in 150 mm tissue‐culture dishes (typically *n* = 18 dishes per preparation) and expanded in serum‐containing medium for at least 48 h. When cultures reached approximately 80%–90% confluence, cells were washed twice with PBS and incubated in serum‐free medium for 24 h before collection. Conditioned medium was cleared by three sequential centrifugation steps at 300 × g for 5 min each at 4°C to remove cells and large debris. The LO‐enriched fraction was then generated from the medium‐speed pellets by centrifuging the cleared conditioned medium at 10 000 × g. The supernatant was subsequently centrifuged at 100 000 × g for 60 min to collect the sEV‐enriched fraction. All ultracentrifugation steps were performed at 4°C using an SW28 swinging‐bucket rotor (Beckman‐Coulter, USA) where applicable. Fresh EV pellets were further resuspended in 0.2 µm‐filtered PBS and loaded at the bottom of Beckman Coulter ultracentrifuge tubes (Cat. #344058). Discontinuous iodixanol gradients were prepared by sequentially layering 30% (4.3 mL; 1.20 g/mL), 25% (3 mL; 1.15 g/mL), 15% (2.5 mL; 1.10 g/mL), and 5% (6 mL; 1.08 g/mL) iodixanol solutions. Gradients were centrifuged at 100 000 × g for 4 h at 4°C. LO fractions were recovered from the 1.10 to 1.15 g/mL density range, whereas sEVs derived from the 100 000 × g pellet were recovered from the 1.10 g/mL density fraction following previously standardized procedures [10]. Purified EVs were washed in 0.2 µm‐filtered PBS by ultracentrifugation at 100 000 × g for 60 min, resuspended in 0.2 µm‐filtered PBS, aliquoted, and stored at −80°C. EV preparation aliquots were used only once after the first thaw and discarded afterward, to avoid repeated freeze–thaw cycles. Unless otherwise stated, EV input for in vitro treatments was normalized to a protein concentration of 20 µg/mL. Dose‐response experiments used the EV concentrations indicated in the relevant figure legends. For in vivo EV education or rescue experiments, animals received 10 µg of EV protein in 100 µL PBS per injection. EV quality was assessed by particle sizing, protein quantification, and immunoblotting for EV‐enriched and negative markers.

### 4.7 Tunable and Microfluidic Resistive Pulse Sensing for Particle Sizing Characterization

EV size distribution and concentration were measured by TRPS using the qNanoGold platform (Izon Science). Freshly purified EVs were diluted 1:40 in 0.2 µm‐filtered PBS. For TRPS, LOs were analyzed using an NP2000 nanopore and sEVs using an NP250 nanopore. Membranes were stretched to 47 mm, and voltage was set to 0.04 V for LOs or 0.5 V for sEVs to obtain a stable baseline current of approximately 120 nA. Particle size and concentration were calibrated using Izon calibration particles (CPC2000 diluted 1:1000 for large EVs and CPC200 diluted 1:100 for sEVs). At least 500 events were recorded per sample at 5 mbar positive pressure.

### 4.8 Enrichment of Circulating LOs From Mouse Plasma

For circulating EV analysis, blood was collected from mice at endpoint into BD Vacutainer K2 EDTA tubes. Samples were processed within 2 h of collection. Platelet‐poor plasma was generated by sequential centrifugation at 500 × g for 10 min and 2000 × g for 20 min. For LO enrichment, approximately 800 µL of plasma was pooled from mice within each experimental group using an equal volume from each animal. Pooled plasma was diluted in 0.2 µm‐filtered PBS to a final volume of 1 mL and centrifuged at 10 000 × g for 30 min at 4°C. Crude plasma LO pellets were resuspended in 10 µL of 0.2 µm‐filtered PBS and used for downstream direct stochastic optical reconstruction microscopy (dSTORM) analysis of PIGS‐ and Caveolin‐1‐positive particles.

### 4.9 Protein Quantification and EV Dosing

Protein concentration in EV preparations and whole‐cell lysates was measured using the Pierce BCA Protein Assay Kit (Thermo Fisher Scientific), according to the manufacturer's instructions. A bovine serum albumin (BSA) standard curve ranging from 20 to 2000 µg/mL was used, and samples were measured with at least three technical replicates. Buffer compatibility with PBS, RIPA, and iodixanol‐containing samples was confirmed before analysis. EV doses for functional assays were normalized to total protein unless otherwise noted.

### 4.10 Whole‐Cell/EV Lysates and Immunoblotting

Whole‐cell lysates were prepared from cultured cells between 80% and 90% confluence. Cell monolayers were washed three times with chilled PBS, scraped, and lysed in RIPA buffer. For EV lysates, after gradient density purification RIPA buffer 10× was added to the EV preparations in PBS to a final 1× concentration for lysis. Lysates were supplemented with Complete protease inhibitor (Roche) and incubated on ice for 20 min, followed by centrifugation at 10 000 × g for 30 min at 4°C. Cleared supernatant was collected, and protein concentration was determined by BCA assay. EV and whole‐cell lysate samples were separated by SDS‐PAGE (1–10 µg of protein) and transferred to nitrocellulose or low‐fluorescence PVDF membranes. Immunoblotting was performed using primary antibodies including HSPA5 (Cell Signaling Technology, #3177, 1:1000), cytochrome c (Cell Signaling Technology, #4280, 1:1000), TSG101 (Santa Cruz Biotechnology, sc‐7964, 1:1000), CD9 (Santa Cruz Biotechnology, sc‐13118, 1:1000), CD81 (Abcam, ab79559, 1:10 000), Caveolin‐1 (Santa Cruz Biotechnology, sc‐894, 1:10 000), cytokeratin 18 (KRT18) (Abcam, ab93741, 1:10 000), STING (D2P2F; Cell Signaling Technology, #13647, 1:1000), SEPT2 (Sigma, HPA018481, 1:1000), beta‐actin (Sigma, 1:2500), and glyceraldehyde 3‐phosphate dehydrogenase (GAPDH) (Cell Signaling Technology, 1:1500). Horseradish peroxidase (HRP)‐conjugated anti‐mouse and anti‐rabbit secondary antibodies (Cell Signaling Technology, 1:5000) and fluorescent StarBright 560 anti‐mouse and StarBright 700 anti‐rabbit secondary antibodies (Bio‐Rad, 1:2500) were used where appropriate.

### 4.11 Transmission Electron Microscopy

Purified EV pellets were fixed with 4% paraformaldehyde (PFA) and post‐fixed in 0.5% osmium tetroxide. Samples were dehydrated through graded ethanol, block stained with 1% uranyl acetate in 50% ethanol for 30 min, embedded in Taab 812 resin, and polymerized overnight at 60°C. Ultrathin sections were imaged using a Hitachi 7100 transmission electron microscope equipped with a Veleta 2000 × 2000 pixel side‐mounted CCD camera; (Olympus).

### 4.12 Super‐Resolution Microscopy and dSTORM of EVs

dSTORM was performed on an ONI Nanoimager system (Oxford Nanoimaging) using a method developed by our group (Newman et al., *under revision*). Purified sEVs and LOs were diluted 1:100 and 1:40 in PBS, respectively, and 100 µL was loaded into µ‐Slide VI 0.5 glass‐bottom chambers (Ibidi). EVs were immobilized by centrifugation at 2000 rpm for 3 min at 4°C, washed twice with PBS, fixed in 4% paraformaldehyde for 10 min, and blocked with 5% BSA for 30 min. Samples were stained for 45 min with the ONI Pan‐EV membrane dye, fluorophore‐conjugated primary antibodies, or unconjugated primary antibodies followed by fluorescent secondary antibodies. Reagents included RPN2‐ alexa fluor (AF)647 (Novus Biologicals; 7 µg/mL), CD81/CD63/CD9‐CF568 (ONI EV Profiler Kit; 1:6.25), CD63‐AF561 (ONI; 1:25), PIGS (Proteintech; 7 µg/mL), Caveolin‐1 (Santa Cruz Biotechnology; 4 µg/mL), dsDNA clone AE‐2 (Merck Millipore; 10 µg/mL), and dsRNA clone J2 (Thermo Fisher Scientific; 5 µg/mL). Unconjugated antibodies were detected with anti‐rabbit IgG‐AF647 (Invitrogen; 5 µg/mL) or anti‐mouse IgG‐CF568 (Sigma–Aldrich; 5 µg/mL). Secondary‐only controls were used to assess nonspecific fluorescence.

For imaging, 100 µL of dSTORM imaging buffer was added to each lane immediately before acquisition. Six fields of view were acquired per lane across consistent lower, middle, and upper regions using an illumination angle of 53°. Raw images were analyzed using ONI CODI software. Localization filtering parameters were defined using secondary‐only controls, and Pan‐EV membrane dye localizations were used to identify membrane‐positive clusters corresponding to individual EVs. Colocalization of protein or nucleic acid markers with membrane‐stained EV clusters was quantified using CODI to determine the percentage of single‐positive and double‐positive EVs. Plasma‐derived LO preparations were analyzed using the same workflow to quantify circulating PIGS‐positive and Caveolin‐1‐positive particles.

To distinguish external from intravesicular nucleic acid signals, LO preparations were analyzed under intact, permeabilized, and nuclease‐treated conditions. Intact LOs were stained with anti‐dsDNA or anti‐dsRNA antibodies to detect external nucleic acid signal, whereas permeabilized LOs were stained to detect both external and intravesicular signal. Permeabilization was performed using the EV Profiler Kit permeabilization buffer (ONI). For nuclease treatments, samples were treated on‐slide with 1 µL DNase I per lane (Invitrogen TURBO DNase; 2 U/µL) for 30 min at 37°C or 10 µL RNase III per lane (New England Biolabs; 2000 U/mL), prepared according to the manufacturer's instructions and incubated for 20 min at 37°C. In intact nuclease‐treated LOs, external nucleic acids were digested before staining, whereas nuclease‐treated LOs that were subsequently washed and permeabilized were used to assess protected intravesicular nucleic acid signal.

### 4.13 Vesicle Flow Cytometry

Single‐particle flow cytometry was used to quantify dsRNA‐positive particles within the LO‐size gate. EV preparations were stained with PE‐conjugated J2 antibody to detect dsRNA and FITC‐conjugated wheat germ agglutinin (WGA) to identify membrane‐bound particles. Samples were incubated for 1 h at room temperature in the presence of 0.1% saponin for permeabilization. Data were acquired on a BD FACSymphony A5 using fluorescence‐triggered detection. Size calibration and gating were performed with Rosetta Calibration Beads (Exometry), and analyses were restricted to particles ≥ 1 µm. WGA‐positive events were used to define vesicular populations, and dsRNA positivity was quantified within this gate. Buffer‐only, single‐stained, and fluorescence‐minus‐one controls were included for gating and compensation. Data were analyzed in FlowJo v11.

### 4.14 BM‐MSC EV Treatments and Conditioned Medium Collection

Human or murine BM‐MSCs were plated; after 24 h of adherence, serum‐free medium was replaced, and cells were starved for 4 h prior to being exposed to purified LOs, sEVs, or PBS/vehicle control for 24 h. Unless otherwise indicated, in vitro EV treatments were performed using 20 µg/mL EV protein. Dose‐response experiments used the EV concentrations indicated in the corresponding figures. For transcriptomic and qPCR analyses, RNA was collected at the time points indicated in each experiment. For conditioned medium experiments, BM‐MSCs were treated with EVs, washed as appropriate, and conditioned medium was collected for cytokine/chemokine measurement, tumor‐cell migration, neutrophil migration, and neutrophil polarization assays. All the experiments were performed at least three times, and each experimental group had at least three biological replicates.

### 4.15 Cytokine, Chemokine, and 2’3’‐cGAMP Detection

Soluble cytokines and chemokines released by EV‐treated BM‐MSCs were measured in conditioned medium using the Human Cytokine/Chemokine Panel A 48‐Plex Discovery Assay Array (HD48A; Eve Technologies), according to the manufacturer's protocol. Briefly, conditioned media were collected from BM‐MSC cultures following treatment with LOs, sEVs, or vehicle control, clarified to remove cellular debris, and stored at −80°C until analysis. Samples were submitted to Eve Technologies for multiplex quantification of cytokines and chemokines. Analyte concentrations were calculated from standard curves generated for each target and reported as concentration per volume of conditioned medium.

2’‐3’‐cyclic GMP‐AMP (2’‐3’‐cGAMP) was quantified using the 2’‐3’‐cyclic GAMP ELISA Kit (Invitrogen), according to the manufacturer's instructions. Donor cancer cells, purified LOs, and sEVs were processed for cGAMP detection using 20 µg of total protein per sample as input for normalization. Absorbance was measured using a microplate reader at the wavelength recommended by the manufacturer, and 2’‐3’‐cGAMP concentrations were calculated from the kit standard curve. Values below the assay detection limit were handled as zero for downstream analysis.

### 4.16 RNA Extraction, RT‐qPCR, and Primer Information

Total RNA was extracted from cells using the Quick‐RNA Miniprep Kit (Zymo Research), according to the manufacturer's instructions. Gene expression was analyzed by one‐step reverse transcription quantitative PCR (RT‐qPCR) using the iTaq Universal One‐Step RT‐qPCR Kit (Bio‐Rad) on a QuantStudio 5 Real‐Time PCR System. Briefly, 40 ng of total purified RNA was added directly to one‐step RT‐qPCR reactions containing reverse transcriptase, universal reaction mix, and gene‐specific primer pairs. Reverse transcription and quantitative PCR amplification were performed in the same reaction according to the manufacturer's recommended cycling conditions. Relative gene expression was normalized to housekeeping genes and calculated using the ΔΔCt method. The following primer pairs were used for inflammatory, interferon‐stimulated, nucleic acid‐sensing, and immunosuppressive genes. Human primers included: CCL2, forward 5’‐AGAATCACCAGCAGCAAGTGTCC‐3’ and reverse 5’‐TCCTGAACCCACTTCTGCTTGG‐3’; CCL5, forward 5’‐CCTGCTGCTTTGCCTACATTGC‐3’ and reverse 5’‐ACACACTTGGCGGTTCTTTCGG‐3’; CXCL1, forward 5’‐AGCTTGCCTCAATCCTGCATCC‐3’ and reverse 5’‐TCCTTCAGGAACAGCCACCAGT‐3’; CXCL8, forward 5’‐GAGAGTGATTGAGAGTGGACCAC‐3’ and reverse 5’‐CACAACCCTCTGCACCCAGTTT‐3’; IL6, forward 5’‐AGACAGCCACTCACCTCTTCAG‐3’ and reverse 5’‐TTCTGCCAGTGCCTCTTTGCTG‐3’; CXCL10, forward 5’‐GGTGAGAAGAGATGTCTGAATCC‐3’ and reverse 5’‐GTCCATCCTTGGAAGCACTGCA‐3’; CXCL11, forward 5’‐AAGGACAACGATGCCTAAATCCC‐3’ and reverse 5’‐CAGATGCCCTTTTCCAGGACTTC‐3’; RSAD2, forward 5’‐CCAGTGCAACTACAAATGCGGC‐3’ and reverse 5’‐CGGTCTTGAAGAAATGGCTCTCC‐3’; ARG1, forward 5’‐TCATCTGGGTGGATGCTCACAC‐3’ and reverse 5’‐GAGAATCCTGGCACATCGGGAA‐3’; IL10, forward 5’‐TCTCCGAGATGCCTTCAGCAGA‐3’ and reverse 5’‐TCAGACAAGGCTTGGCAACCCA‐3’; and TNF, forward 5’‐CTCTTCTGCCTGCTGCACTTTG‐3’ and reverse 5’‐ATGGGCTACAGGCTTGTCACTC‐3’. Murine primers included: Ccl5, forward 5’‐CCTGCTGCTTTGCCTACCTCTC‐3’ and reverse 5’‐ACACACTTGGCGGTTCCTTCGA‐3’; Cxcl10, forward 5’‐ATCATCCCTGCGAGCCTATCCT‐3’ and reverse 5’‐GACCTTTTTTGGCTAAACGCTTTC‐3’; Rsad2, forward 5’‐GGAAGGTTTTCCAGTGCCTCCT‐3’ and reverse 5’‐ACAGGACACCTCTTTGTGACGC‐3’; Irf3, forward 5’‐CGGAAAGAAGTGTTGCGGTTAGC‐3’ and reverse 5’‐CAGGCTGCTTTTGCCATTGGTG‐3’; Irf7, forward 5’‐CCTCTGCTTTCTAGTGATGCCG‐3’ and reverse 5’‐CGTAAACACGGTCTTGCTCCTG‐3’; Ifna1, forward 5’‐GGATGTGACCTTCCTCAGACTC‐3’ and reverse 5’‐ACCTTCTCCTGCGGGAATCCAA‐3’; and Ddx58/Rig‐I, forward 5’‐AGCCAAGGATGTCTCCGAGGAA‐3’ and reverse 5’‐ACACTGAGCACGCTTTGTGGAC‐3’. Housekeeping gene primers included human GAPDH, forward 5’‐GTCTCCTCTGACTTCAACAGCG‐3’ and reverse 5’‐ACCACCCTGTTGCTGTAGCCAA‐3’; mouse Gapdh, forward 5’‐CATCACTGCCACCCAGAAGACTG‐3’ and reverse 5’‐ATGCCAGTGAGCTTCCCGTTCAG‐3’; human ACTB, forward 5’‐CACCATTGGCAATGAGCGGTTC‐3’ and reverse 5’‐AGGTCTTTGCGGATGTCCACGT‐3’; and mouse Actb, forward 5’‐CATTGCTGACAGGATGCAGAAGG‐3’ and reverse 5’‐TGCTGGAAGGTGGACAGTGAGG‐3’. The housekeeping gene used for each experiment was selected according to the species of the biological sample.

### 4.17 RNA Sequencing and Transcriptomic Analysis of EV‐Treated BM‐MSCs

RNA‐seq was performed using three biological replicates per group. RNA was extracted from experimental samples, treated with TURBO DNase (Thermo Fisher Scientific, AM2238), and cleaned using the Zymo RNA Clean and Concentrator kit (Zymo Research, R1014). RNA concentration and quality were assessed using RiboGreen reagent (Thermo Fisher Scientific, R11490) and Agilent TapeStation RNA ScreenTape reagents (5067‐5579). 2.5 ng of RNA was used for library preparation with the SMARTer Pico V2 kit (Takara, 634412). Libraries were quantified using KAPA Library Quantification Kits (Roche, KK4824) and sequenced as single‐end reads on an Illumina NovaSeq 6000.

Transcripts were quantified using Salmon, and differential expression analysis was performed with DESeq2. Heatmaps were generated using the ComplexHeatmap R/Bioconductor package on scaled log‐expression values (z‐scores), with Euclidean distance and Ward linkage implemented with the fastcluster package. For selected heatmaps, genes with absolute log2 fold change >1 were ranked by *p*‐value, and the top genes were plotted. GSEA was performed on genes ranked by fold change using clusterProfiler and MSigDB gene‐set collections. Significantly enriched gene sets were reported using Benjamini‐Hochberg adjusted *p*‐values < 0.05.

### 4.18 Innate Immune Reporter Assays and Pathway Perturbation

Innate immune activation by LOs was evaluated using a BM‐MSC reporter‐cell assay based on secreted embryonic alkaline phosphatase (SEAP) activity. BM‐MSCs were transfected with the pNiFty2‐56K‐SEAP plasmid (InvivoGen) using Lipofectamine Stem Transfection Reagent. The plasmid was first propagated in Mix & Go! DH5α competent cells, and bacterial clones were selected with zeocin at 25 µg/mL. Following transfection, Zeocin‐resistant BM‐MSCs were selected using zeocin at 100 µg/mL to generate reporter BM‐MSCs. Reporter activity was measured using QUANTI‐Blue SEAP Detection Reagent (InvivoGen), according to the manufacturer's instructions. For reporter assays, BM‐MSC reporter cells were plated in 96‐well plates at 5 × 10^3^ cells per well and allowed to adhere for 24 h. The medium was then replaced with serum‐free medium before treatment. Cells were exposed to LOs at 20 µg/mL. Poly(I:C) and poly(dA:dT) complexed with transfection reagent LyoVec (Invivogen) were used as positive controls for nucleic acid‐induced innate immune activation and were added at the same time as LOs at final concentrations of 1 µg/mL and 10 µg/mL, respectively. To assess STING‐dependent signaling, reporter BM‐MSCs were pretreated with the STING inhibitor H‐151 (MedChemExpress) at a final concentration of 15 µM. H‐151 was added 4 h before LO, poly(I:C), or poly(dA:dT) exposure and was maintained in the culture medium throughout LO or agonist stimulation. Thus, STING signaling was inhibited before and during exposure to EV‐associated cargo. DMSO was used as the vehicle control at the same volume used in inhibitor‐treated groups. A toxicity curve was performed to select inhibitor conditions that did not induce detectable cell death.

To evaluate whether endosomal acidification or degradation influenced LO‐induced inflammatory signaling, BM‐MSCs were treated with Bafilomycin A1 (MedChemExpress) at a final concentration of 100 nM. Bafilomycin A1 was added 2 h before LO exposure and maintained in the medium during LO stimulation (24 h). DMSO was used as the vehicle control at the same volume used in the Bafilomycin A1‐treated group. For both studies, LO‐induced inflammatory responses following pathway perturbation were measured by either SEAP reporter activity and/or downstream gene‐expression readouts, as described in the RT‐qPCR section.

### 4.19 Generation of NanoLuc/GFP Reporter 4T1.2 Cells

NanoLuc/GFP reporter‐expressing 4T1.2 cells were generated by lentiviral transduction. Lentiviral particles were produced in HEK293T cells by co‐transfection of pLenti‐PalmGRET (Addgene #158221, gift from Dr Charles P. Lai) with packaging plasmids psPAX2 (Addgene #12260) and pMD2.G (Addgene #12259) using TurboFectin 8.0. Viral supernatants were collected at 48 and 72 h post‐transfection, filtered through a 0.45 µm filter, and stored at −80°C. 4T1.2 cells were transduced with lentiviral supernatant in the presence of 8 µg/mL polybrene overnight. GFP‐positive cells were enriched by bulk FACS sorting, and the top 20% brightest GFP‐positive cells were collected, expanded, and selected with puromycin (5 µg/mL).

### 4.20 Immunofluorescence Microscopy of Cells

Cellular immunofluorescence was used to visualize bleb formation in the plasma membrane and dsRNA accumulation/localization. Cells seeded in round cover class, after 24 h cells were fixed and permeabilized using BD Cytofix/Cytoperm Fixation/Permeabilization Solution Kit, and stained with dsRNA J2 antibody (Jena Bioscience, rabbit‐raised, 1:100 for cell imaging), rhodamine‐phalloidin (Cytoskeleton Inc., #PHDR1, 100 nM), WGA‐AF488 or WGA‐AF555 (Invitrogen, #W11261/#W32464, 1 µg/mL), and DAPI (Invitrogen, #62248, 1 µg/mL) across different experiments. Cells were visualized in a Leica Stellaris 8‐STED Super‐resolution Confocal Microscope.

### 4.21 Tumor‐Cell Migration and Dynamic Bone‐on‐Chip Assay

Cancer cell and neutrophil migration toward conditioned medium from EV‐treated BM‐MSCs was evaluated using transwell migration assays. BM‐MSCs were treated with LOs, sEVs, or vehicle control, and conditioned medium was collected as described above. PC3 cells or freshly isolated human neutrophils were seeded into the upper chamber of Transwell inserts containing 8.0 µm pore‐size PET membranes. Conditioned medium from control or EV‐treated BM‐MSCs was placed in the lower chamber as the chemoattractant. After migration, non‐migrated cells were removed from the upper surface of the membrane, and migrated cells on the lower surface were fixed, stained with crystal violet, and quantified by counting the number of migrated cells per insert or imaging field.

For dynamic 3D bone‐on‐a‐chip assays, we used a previously described PDMS microfluidic platform that supports BM‐MSC culture under continuous perfusion and microscopic imaging [50]. Chips were coated with laminin, and BM‐MSCs were seeded at 1 × 10^6^ cells/mL in 50 µL. After 3–6 h, non‐adherent cells were removed by flushing, and flow was initiated 24 h later at 30 µL/h using a syringe pump. LOs or vehicle were then circulated through the chip to condition the stromal compartment. PC3 cells were subsequently introduced into the circulating medium, and their recruitment and attachment to the BM‐MSC‐seeded matrix were quantified by imaging as the number of matrix‐associated cells per field or region of interest. This system enabled assessment of LO‐mediated stromal education under physiologically relevant flow, matrix, and shear conditions.

### 4.22 CRISPR‐Cas9 Knockout of STING and SEPT2

CRISPR‐Cas9 knockout of human STING/TMEM173 in BM‐MSCs and mouse Sept2 in 4T1.2 cells was performed by ribonucleoprotein (RNP) delivery using the Alt‐R CRISPR‐Cas9 system (Integrated DNA Technologies) and Lipofectamine CRISPRMAX (Thermo Fisher Scientific), according to the manufacturer's recommendations. Gene‐specific crRNAs were annealed with tracrRNA (1:1 molar ratio) in Nuclease‐Free Duplex Buffer (Integrated DNA Technologies) to generate duplex guide RNAs, and incubated at 95°C for 5 min followed by a continuous ramp‐down temperature step of −1°C/min. Annealed gRNAs were complexed with RFP‐SpCas9 recombinant protein before transfection (1.2:1 molar ratio, respectively). The TMEM173‐targeting crRNA sequence was 5'‐GCUGGGACUGCUGUUAAACGGUUUUAGAGCUAUGCU‐3. The Sept2‐targeting crRNA sequence was 5'‐UAGUGGACACUCCCGGCUACGUUUUAGAGCUAUGCU‐3. A scrambled sequence not targeting any sequence on the human or murine genome was used as a negative control: 5'‐CGUUAAUCGCGUAUAAUACGGUUUUAGAGCUAUGCU‐3. RFP‐positive cells were isolated by flow sorting approximately 24 h after transfection. STING knockout BM‐MSCs were enriched by bulk sorting, whereas 4T1.2 Sept2‐edited cells were sorted as single cells to generate monoclonal populations. Knockout was validated by western blotting.

### 4.23 Flow Cytometry and High‐Dimensional Analysis

Flow cytometry was used to assess neutrophil polarization in vitro and to profile bone marrow immune populations in vivo. For human neutrophil polarization assays, freshly isolated human neutrophils were exposed for 1–6 h to conditioned medium collected from BM‐MSCs treated with LOs, sEVs, or vehicle control. After stimulation, cells were stained with LOX‐1‐FITC (Thermo Fisher Scientific, 1:100), CD66b‐PE (Thermo Fisher Scientific, 1:100), and CD62L‐AF647 (1:50) to evaluate acquisition of a PMN‐MDSC‐like phenotype.

For in vivo immune profiling, bone marrow cells were harvested from experimental mice, filtered into single‐cell suspensions, subjected to red blood cell lysis with ACK lysis buffer, counted, and stained for spectral flow cytometry. Cells were processed using the BD Cytofix/Cytoperm kit according to the manufacturer's recommendations for fixation and permeabilization/blocking prior to acquisition. The staining panel included CD45‐PE‐Cy7 (BD, 1:100), CD3‐PE‐Cy5 (BD, 1:100), CD11b‐BB700 (BD, 1:200), CD11c‐BV421 (BD, 1:200), F4/80‐BUV496 (BD, 1:200), Ly6C‐FITC (BD, 1:100), Ly6G‐BV563 (BD, 1:200), CD14‐APC (BD, 1:200), CD16/32‐BUV737 (BD, 1:200), MHC‐II‐BV605 (BD, 1:200), B220‐PerCP (BD, 1:200), CD177‐AF647 (BD, 1:200), PD‐L1‐BV711 (BD, 1:100), CD49d‐BV786 (BD, 1:200), CD86‐BV510 (BD, 1:200), CXCR2‐BUV661 (BD, 1:100), CXCR4‐PE (BD, 1:100), TREM‐1‐BUV395 (BD, 1:200), CD64‐BV650 (BD, 1:200), CD62L‐BUV805 (BD, 1:200), Siglec‐F‐BV750 (BD, 1:200), and BD Horizon Fixable Viability Stain 780 (1:1000) [51, 52, 53]. Samples were acquired on a Sony ID7000 spectral flow cytometer. Autofluorescence was defined using unstained controls and removed during spectral unmixing. Single‐stained reference controls for each fluorophore were used to generate spectral reference signatures and perform unmixing. A total of 15 000 events were acquired. Gating was performed in FlowJo v10 after exclusion of debris, doublets, and dead cells, followed by biexponential transformation of fluorescence parameters. Neutrophils and PMN‐MDSC‐like populations were identified using a sequential gating strategy that included live cells, CD45^+^ leukocytes, CD11b^+^ myeloid cells, Ly6G^+^ neutrophils, and additional phenotypic markers including Ly6C, CD14, PD‐L1, CXCR2, MHC‐II, CD62L, CD177, and related myeloid markers [53]. For high‐dimensional analysis, live cells from five mice were concatenated and exported as a single FCS file. Dimensionality reduction visualization was performed using the Uniform Manifold Approximation and Projection (UMAP) algorithm. FlowSOM clustering [54] was then applied and overlaid onto the UMAP projection to objectively identify phenotypically distinct populations based on marker‐expression patterns. Metaclusters with similar expression profiles were manually merged when appropriate and annotated according to the expression of defining markers.

### 4.24 Scaffold and Intratibial Neutrophil Recruitment Assays

For in vivo neutrophil recruitment assays, mouse bone marrow cells or MSCs were seeded either into 24‐well plates or into 3D scaffold disks. Scaffolds consisted of reticulated polycarbonate polyurethane urea matrix disks (5 mm × 2 mm; CS1‐0502‐25, Biomerix Corp/DSM Biomedical). Cells were seeded at 1 × 10^5^ cells per scaffold or per well and allowed to adhere for at least 24 h [39]. Cells were then treated with EVs for 24 h. EV‐conditioned scaffolds were implanted subcutaneously into C57BL/6 mice flanks, whereas EV‐conditioned cells from wells were injected directly into tibiae. Scaffolds and bone marrow cells were harvested 48 h later, dissociated, and analyzed by flow cytometry for neutrophil recruitment (CD45^+^ Cd11b^+^ Ly6C^neg^ Ly6G^+^).

### 4.25 SEPT2 Knockout Tumor Model and LO Rescue

To test whether tumor‐derived LO production is required for bone marrow PMN‐MDSC accumulation, female 6‐8‐week‐old BALB/c mice were orthotopically implanted in the lower right mammary fat pad with 2 × 10^5^ scramble control or Sept2 knockout 4T1.2 cells (*n* = 5 mice/group). Tumor growth was monitored during the 2‐week experimental period. In rescue cohorts, mice bearing Sept2 knockout or scramble control tumors received systemic LO administration concomitantly with primary tumor growth, consisting of four retro‐orbital injections of 10 µg purified syngeneic LO protein in 100 uL PBS over the 2‐week period, as described in the Orthotopic 4T1.2 tumor education and metastasis model section. At endpoint, plasma and bone marrow were collected for circulating LO dSTORM and spectral flow cytometry analysis.

### 4.26 Tumor‐Bearing vs. Naive Bone Marrow Immune Profiling

To evaluate bone marrow immune remodeling during tumor progression, female 6–8‐week‐old BALB/c mice were orthotopically implanted with 2 × 10^5^ nLuc‐GFP 4T1.2 cells into the lower right mammary fat pad and compared with naive controls. Tumors were followed for 4 weeks (28 days), after which bone marrow and plasma were collected. Circulating plasma EVs were analyzed by dSTORM for PIGS‐positive particles, and bone marrow cells were analyzed by high‐dimensional spectral flow cytometry. GFP‐positive tumor cells in the bone marrow were assessed to determine whether immune remodeling occurred before overt metastatic colonization.

### 4.27 IVIS Imaging and Metastatic Burden Quantification

Bioluminescence imaging was used to monitor metastatic progression in mice injected with GFP/NanoLuc reporter 4T1.2 cells. Animals were anesthetized before imaging and imaged longitudinally using an IVIS system. Nano‐Glo Fluorofurimazine i*n vivo* Substrate (Promega) was injected intraperitoneally (0.5 µM), and Signal intensity was quantified as total bioluminescence flux within defined regions of interest, and focal lesions were counted using predefined thresholds based on autofluorescence signal.

### 4.28 Statistical Analysis

Statistical analyses were performed using GraphPad Prism 9 and/or R 4.6. Data are presented as mean ± Standard Error Mean or as otherwise indicated in the figure legends. Two‐group comparisons were performed using parametric or nonparametric tests as appropriate, and multiple‐group comparisons were analyzed using ANOVA or nonparametric alternatives with post hoc correction. Longitudinal tumor growth or IVIS measurements were analyzed using repeated‐measures or mixed‐effects models when appropriate. Correlations were assessed using Pearson or Spearman tests based on Gaussian distribution characteristics of the sample. RNA‐seq analyses used Benjamini‐Hochberg adjustment for multiple testing, with adjusted *p* < 0.05 considered significant unless otherwise specified. The definition of n corresponds to mice, biological replicates, independent EV preparations, or human donors as specified in each figure legend.

## Author Contributions

**Taylon Felipe Silva**: conceptualization, investigation, funding acquisition, Writing – original draft, methodology, validation, visualization, Writing – review and editing, data curation, project administration, formal analysis, software. **Virginia Marcia Concato‐lopes**: methodology, investigation, data curation, validation, formal analysis. **Fatima Bedier**: methodology, software, data curation, investigation, validation, formal analysis. **Lauren Newman**: methodology, software, data curation, investigation, validation, formal analysis. **Diana Kitka**: data curation, investigation, methodology, validation, formal analysis. **Jamelle Brown**: methodology, investigation, validation, formal analysis. **Blandine Victor**: methodology, investigation, validation, formal analysis. **Minhyung Kim**: methodology, software, data curation, formal analysis. **Tatyana Vagner**: methodology, software, data curation, investigation, validation, formal analysis. **Catherine Grasso**: software, data curation, formal analysis. **Dmitriy Sheyn**: methodology, validation, investigation, formal analysis. **Sungyong You**: software, methodology, investigation, data curation, formal analysis, writing – review and editing. **Michael R. Freeman**: resources, writing – review and editing, conceptualization. **Fayyaz S. Sutterwala**: conceptualization, methodology, investigation, writing – review and editing. **Helen S. Goodridge**: methodology, investigation, writing – review and editing. **Caroline Jefferies**: methodology, investigation, validation, writing – review and editing. **Paola de Candia**: conceptualization, methodology, software, formal analysis, writing – review and editing. **Jlenia Guarnerio**: conceptualization, methodology, investigation, validation, writing – review and editing. **Dolores Di Vizio**: conceptualization, methodology, data curation, investigation, supervision, funding acquisition, visualization, project administration, resources, writing – review and editing.

## Funding

This work was funded by the National Institutes of Health (R01CA218526 and R01CA234557 to D.D.V.; R21CA299812 to T.F.S. and J.G.; and 2R01CA220327 and 1R01CA271750 to M.R.F.), Prostate Cancer Foundation Challenge Award (to M.R.F.), and the Department of Defense Prostate Cancer Research Program (PC240684 to T.F.S.).

## Conflicts of Interest

The authors declare no conflicts of interest.

## Supporting information




**Supporting File 1**: advs78406‐sup‐0001‐SuppMat.pdf.

**Supporting File 2**: advs78406‐sup‐0002‐SuppMat.csv.

## Data Availability

The data that support the findings of this study are openly available in GEO at https://www.ncbi.nlm.nih.gov/geo/, reference number GSE341387.
