# Supplementary material for: Large Oncosomes Induce Pro‐Metastatic Stromal‐Myeloid Reprogramming
Source: Adv Sci (Weinh). 2026 Oct 25:e78406. Online ahead of print. doi: 10.1002/advs.78406 (PMC13696780; doi:10.1002/advs.78406)
Supplement: Supplementary file 1 [file ADVS-9999-e78406-s002.pdf]

SUPPORTING INFORMATION

Large oncosomes induce pro-metastatic stromal-myeloid reprogramming

Taylon Felipe Silva, Virginia Marcia Concato-Lopes, Fatima Bedier, Lauren Newman, Diana Kitka, Jamelle Brown, Blandine Victor, Minhyung Kim, Tatyana Vagner, Catherine Grasso, Dmitriy Sheyn, Sungyong You, Michael R. Freeman, Fayyaz S. Sutterwala, Helen S. Goodridge, Caroline Jefferies, Paola de Candia, Jlenia Guarnerio, Dolores Di Vizio.

Supplementary figures and figure legends

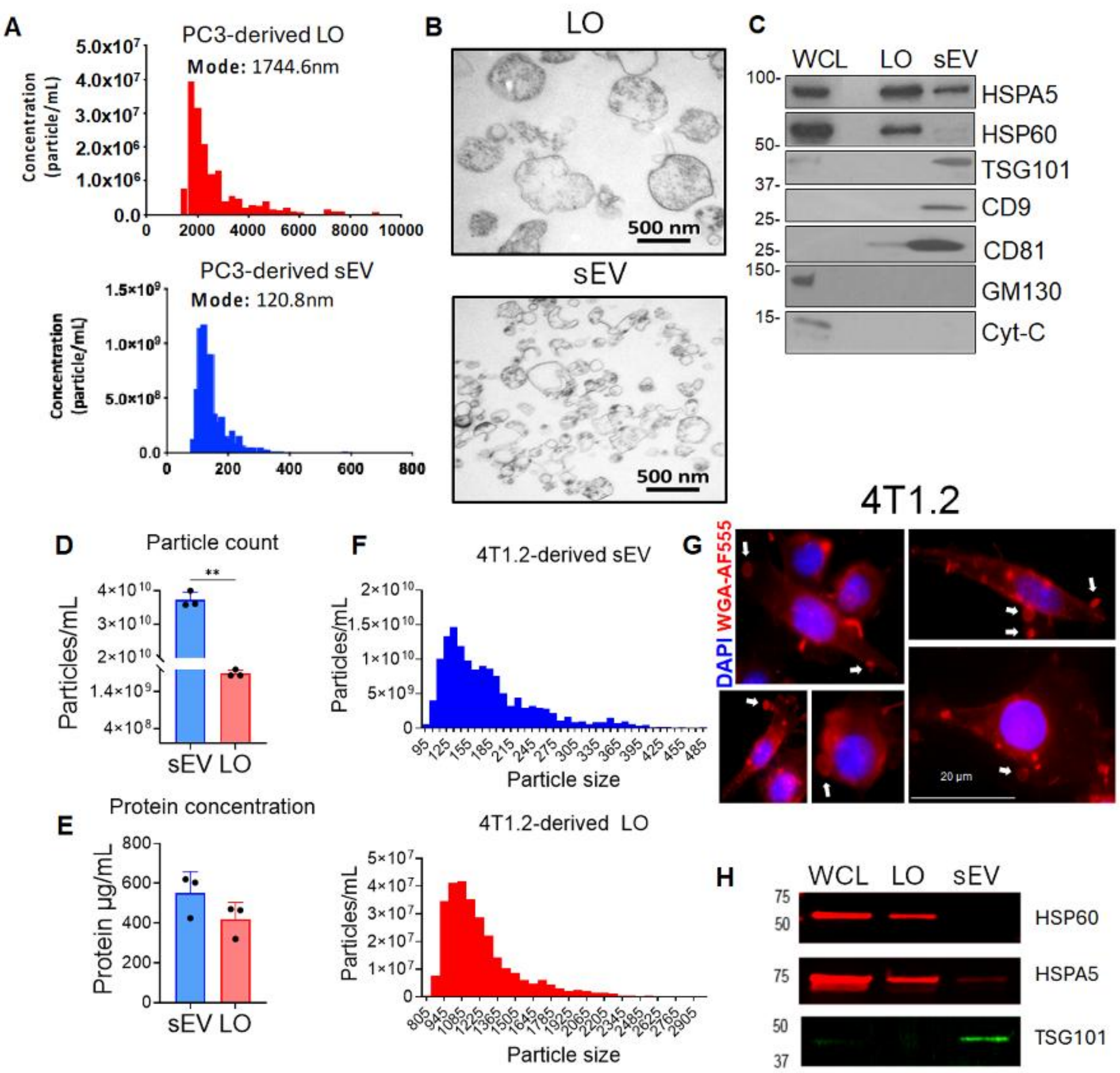

Supplementary Figure 1 - Characterization of large oncosomes and small EVs across cell lines. A, Tunable-resistive pulse sensing (TRPS) size-distribution histograms of PC3-derived LO (top) and sEV (bottom). **B**, Transmission electron microscopy of PC3-derived LO and sEV preparations. **C**, Western blot analysis of PC3 EVs showing LO markers (HSP60, HSPA5), sEV markers (TSG101, CD9, CD81) and cell-associated markers (GM130 and cytochrome c) using HRP-conjugated secondary antibodies. **D**, TRPS particle concentration measurements of 4T1.2-derived LO and sEV. **E**, Total protein abundance of 4T1.2-derived LO and sEV preparations. **F**, TRPS size-distribution histograms of 4T1.2-derived sEV (top) and LO (bottom). **G**, Fluorescence imaging of 4T1.2 bone-metastatic breast cancer cells showing LO shedding. Plasma membrane labeled with WGA-AF555 (red) and nuclei with DAPI (blue). Scale bar, 20 µm. **H**, Fluorescent Western blot of 4T1.2-derived EVs and whole-cell lysate showing LO markers (HSP60, HSPA5) and the sEV marker TSG101 using secondary antibodies conjugated with StarBright Blue 520 in green (anti-mouse) or StarBright Blue 700 in red (anti-rabbit).

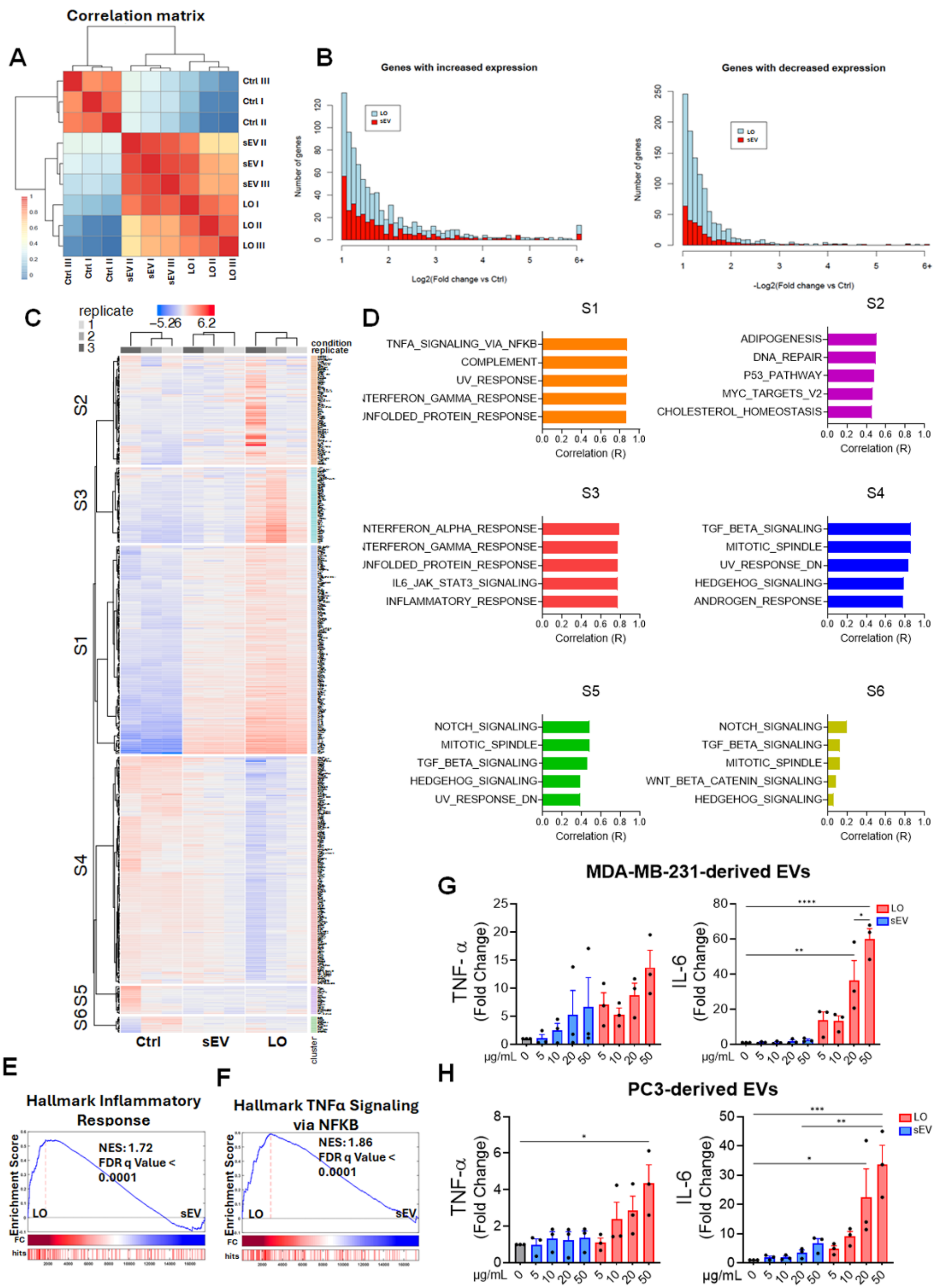

Supplementary Figure 2. RNA-seq quality control and pathway analysis of EV-treated human BM-MSCs. A-B, RNA-seq quality control analyses, including sample-to-sample Pearson correlation matrix (**A**) and distributions of gene

expression changes and concordance/discordance of fold-change directionality between LO and sEV treatments (**B**). **C**, 2-way unsupervised hierarchical clustering analysis of EV-treated BM-MSC RNAseq. The tree-like dendrogram shows the distance between features and the approximate groups. The column annotations show the correlation with the phenotypes. **D**, top 5 HALLMARK gene set pathways enriched in each cluster identified in panel C. **E-F**, GSEA enrichment plots for inflammatory response (**E**) and TNFα signaling via NF-κB (**F**) in LO-treated versus sEV-treated human BM-MSCs. **G-H**, RT–qPCR analysis of IL6 and TNFA expression in human BM-MSCs treated with increasing concentrations of MDA-MB-231-derived (**G**) or PC3-derived (**H**) LO or sEV. Expression was normalized to ACTB. Each dot represents one independent experiment. For all in vitro experiments, data represents at least three independent experiments with at least three biological and three technical replicates. Statistical analyses are described in the Methods.

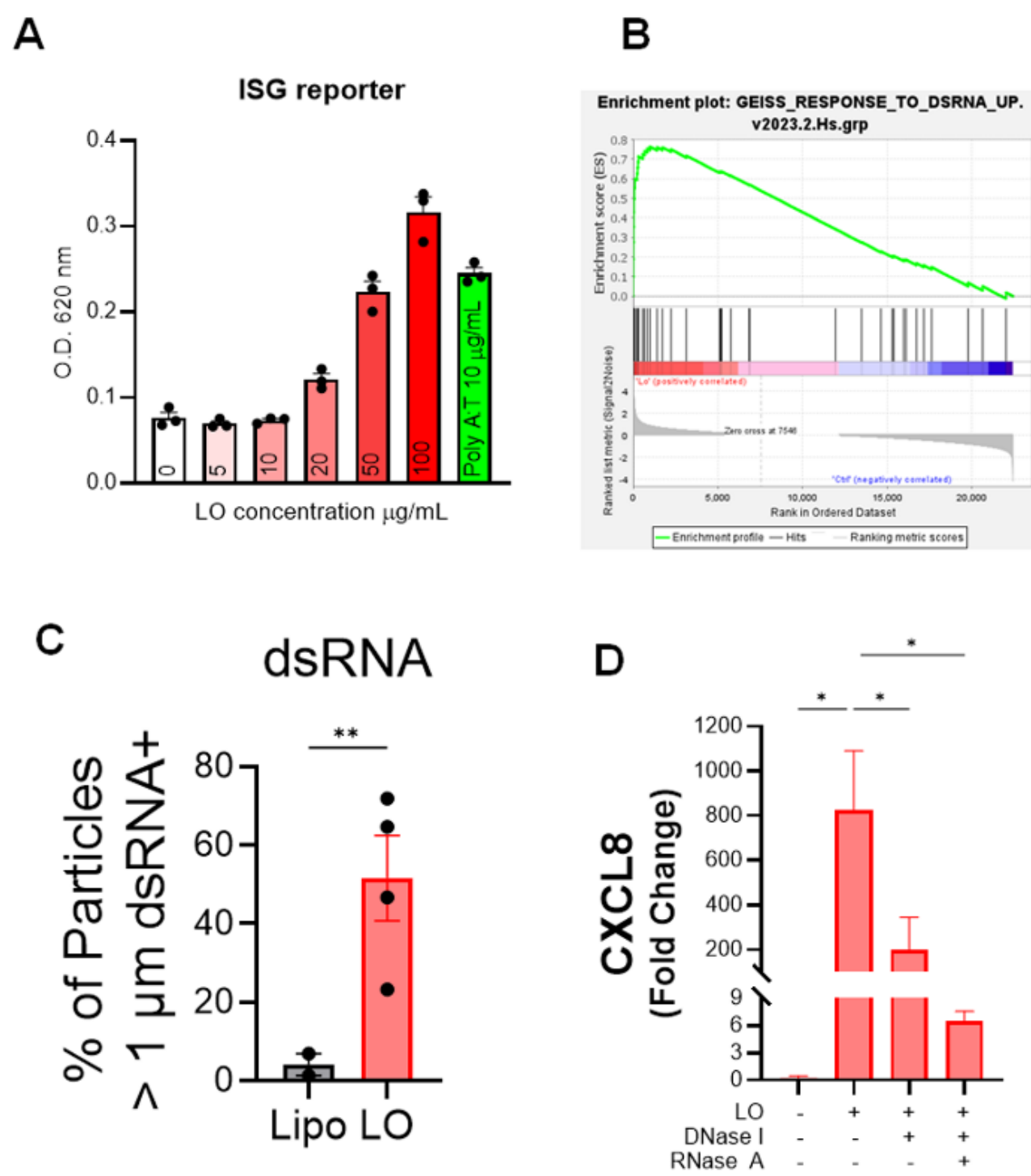

Supplementary Figure 3. Validation and extended analyses of LO-mediated nucleic acid sensing. A, BM-MSC cells expressing secretable alkaline phosphatase (SEAP) under the minimal promoter of Interferon Stimulated Genes (ISG) as reporter system. Treatment with increasing LO concentrations showing correlative effect on the activity of the reporter. Poly(dA:dT) was included as a positive control. **B**, GSEA analysis of GEISS Response to dsRNA upregulation in LO vs ctrl-treated BM-MSC**. C**, Flow cytometry analysis of dsRNA-positive LO and 2 µm liposomes (Lipo) formulated with cholesterol, sphingomyelin, phosphatidylcholine, phosphatidylserine, phosphatidylethanolamine, and PEG2000-conjugated distearoylphosphatidylethanolamine (PEG2000-DSPE) at a molar ratio of 25:7:40:10:15:3, respectively**. D**, RT–qPCR analysis of CXCL8 expression in human BM-MSCs treated with LO pre-incubated with DNase I or/and RNases. Expression was normalized to *ACTB*.

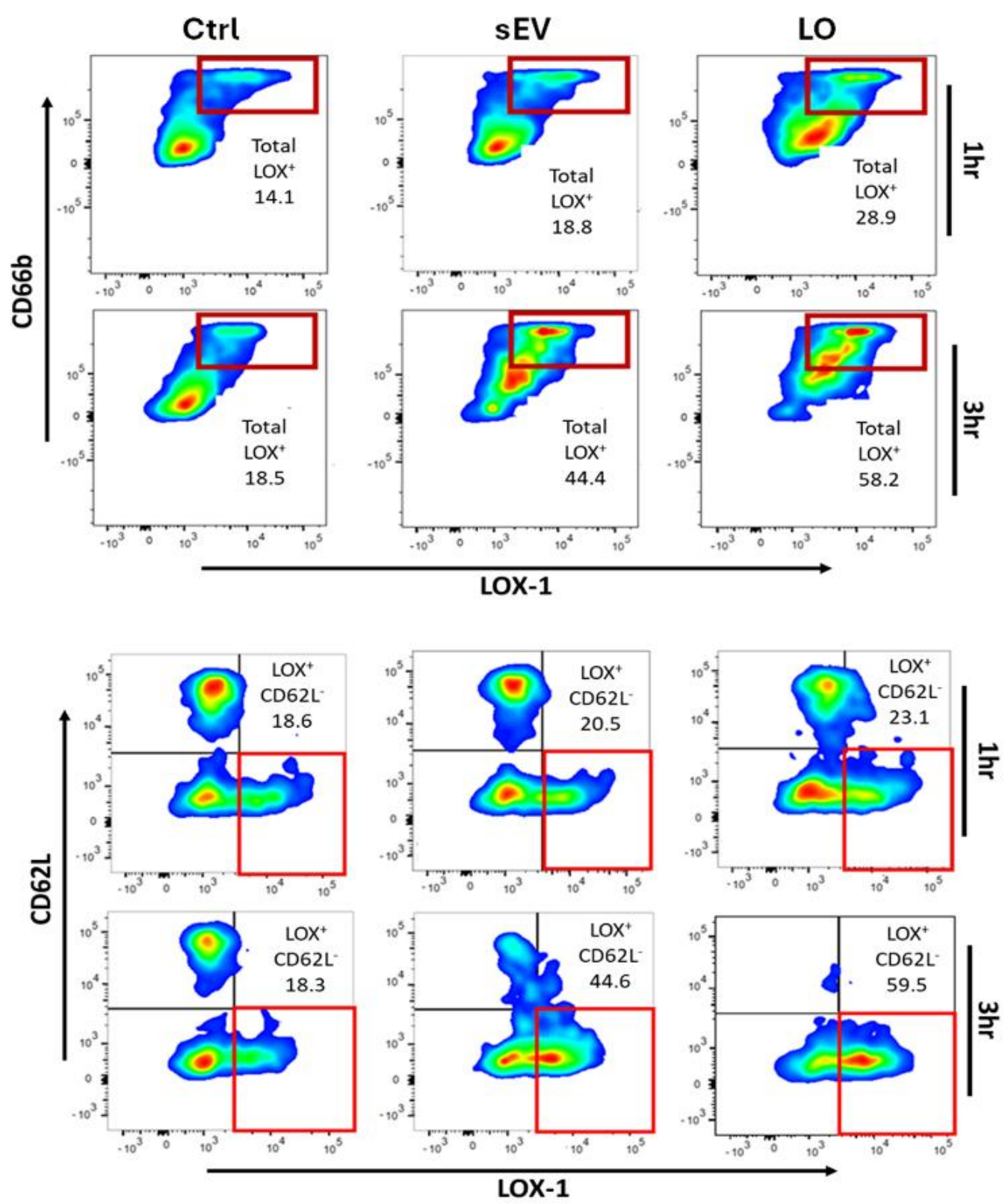

Supplementary Figure 4. LO-treated BM-MSCs induce fast neutrophil polarization. Flow cytometry analysis of CD62L, CD66b, and LOX-1 expression in primary human neutrophils following exposure to conditioned medium from vehicle-, sEV- or LO-treated BM-MSCs at 1 and 3 h, associated with **Figure 4H-I.**

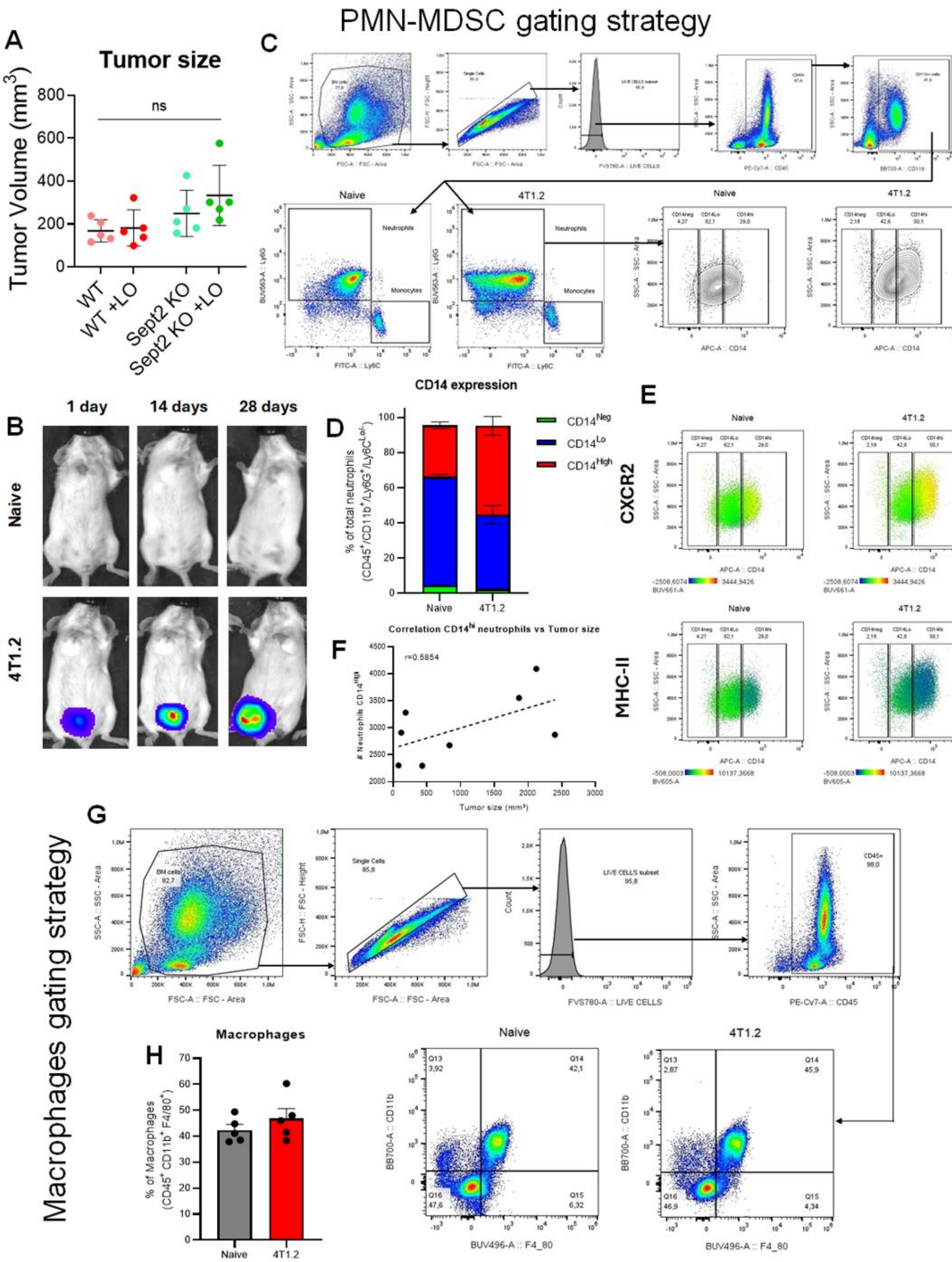

Supplementary Figure 5. Tumor tracking and gating strategies. A, tumor size measurements from experiment described in **Figure 5A**. **B**, Representative IVIS images of tumor growth in BALB/c mice orthotopically implanted with 4T1.2-Luc/GFP cells at days 1, 14 and 28 after implantation. Associated with **Figures 5G-K** and **S5C-G. C**, Gating strategy for identification of neutrophils and PMN-MDSCs based

on CD45, CD11b, Ly6C, Ly6G and CD14 expression. **D**, Quantification of $CD14^-$, $CD14^{lo}$ and $CD14^{hi}$ neutrophils in bone marrow from naïve and tumor-bearing BALB/c mice (n = 5 mice per group). **E**, Expression of CXCR2 (top) and MHC class II (bottom) across neutrophil populations with different CD14 expression level identified by spectral flow cytometry. **F**, Correlation between tumor size and frequency of $CD14^{hi}$ neutrophils in tumor-bearing mice. **G**, Gating strategy and quantification of macrophage populations in bone marrow from naïve and tumor-bearing mice. **H**, Quantification of macrophage populations in **G**.

Supplementary Table Legends

Supplementary Table 1. Gene expression count matrix from RNA-seq of BM-MSCs treated with large oncosomes or small extracellular vesicles. Gene-level RNA-seq counts from bone marrow mesenchymal stem cells (BM-MSCs) treated with PC3-derived large oncosomes (LOs) or small extracellular vesicles (sEVs) for 24 h. Rows represent individual genes and columns represent biological samples used for downstream differential gene expression analyses.
